# The opportunity for sexual selection and the evolution of non-responsiveness to pesticides, sterility inducers and contraceptives

**DOI:** 10.1016/j.heliyon.2018.e00943

**Published:** 2018-11-29

**Authors:** Stephen M. Shuster, Brandy Pyzyna, Loretta P. Mayer, Cheryl A. Dyer

**Affiliations:** a Department of Biological Sciences, Northern Arizona University, Flagstaff, AZ 86011-5640, USA; b Senestech, Inc., 3140 N Caden Court, Suite #1, Flagstaff, AZ 86004, USA

**Keywords:** Biotechnology, Ecology, Evolution, Mathematical biosciences

## Abstract

We illustrate a method for delaying and possibly eliminating the evolution of non-responsiveness to the treatments now used to control pest populations. Using simulations and estimates of the variance in relative fitness, i.e., the opportunity for selection, in a rat-like mammal, we show that the selection responsible for the evolution of non-responsiveness to pesticides and sterility-inducers, is similar in its action to sexual selection, and for this reason can be orders of magnitude stronger than that which exists for untreated populations. In contrast, we show that when contraceptives are used to reduce the fertility of a pest species, with non-responders embedded within such populations, the opportunity for selection favoring non-responsiveness is reduced to that which is expected by chance alone. In pest species with separate sexes, we show that efforts to control pest populations or to mitigate selection favoring non-responsiveness, are likely to be ineffective when members of one sex are sterilized or killed. We also show that while mating preferences can impede the rate at which resistance evolves, they are more likely to accelerate this process, arguing against the use of sterile male approaches for controlling pests. Our results suggest that contraceptives are more effective at controlling pest populations and slowing the evolution of non-responsiveness than treatments that cause sterilization or death in target species. Furthermore, our results indicate that contraceptives that work differentially on each sex will be most effective in mitigating selection favoring non-responders. Our results have significant implications for the development and application of treatments to manage pests, now and into the future.

## 1 Introduction

Human populations and the nutritional resources that support them have undergone unprecedented expansion since the mid-20th century (Tilman et al., 2001; Jackson et al., 2001; Kraussman et al., 2013). Much of this growth can be attributed to the development of pesticides designed to eliminate agricultural and other human-associated pests (Hoy, 1998; Palumbi, 2001). Despite this apparent progress, the removal of large portions of pest populations by chemical means is now known to favor the evolution of pesticide “resistance,” the general term used to describe increasing tolerance by pest species to the chemical substances designed to control them (Greaves and Ayers, 1967; Palumbi, 2001; Diaz et al., 2010). When resistance appears, increasing concentrations or combinations of treatments are required to maintain established levels of pests, often leading to further increases in pesticide resistance within the targeted populations (Leslie et al., 2005; Ishizuka et al., 2008).

**Fig. 1:**
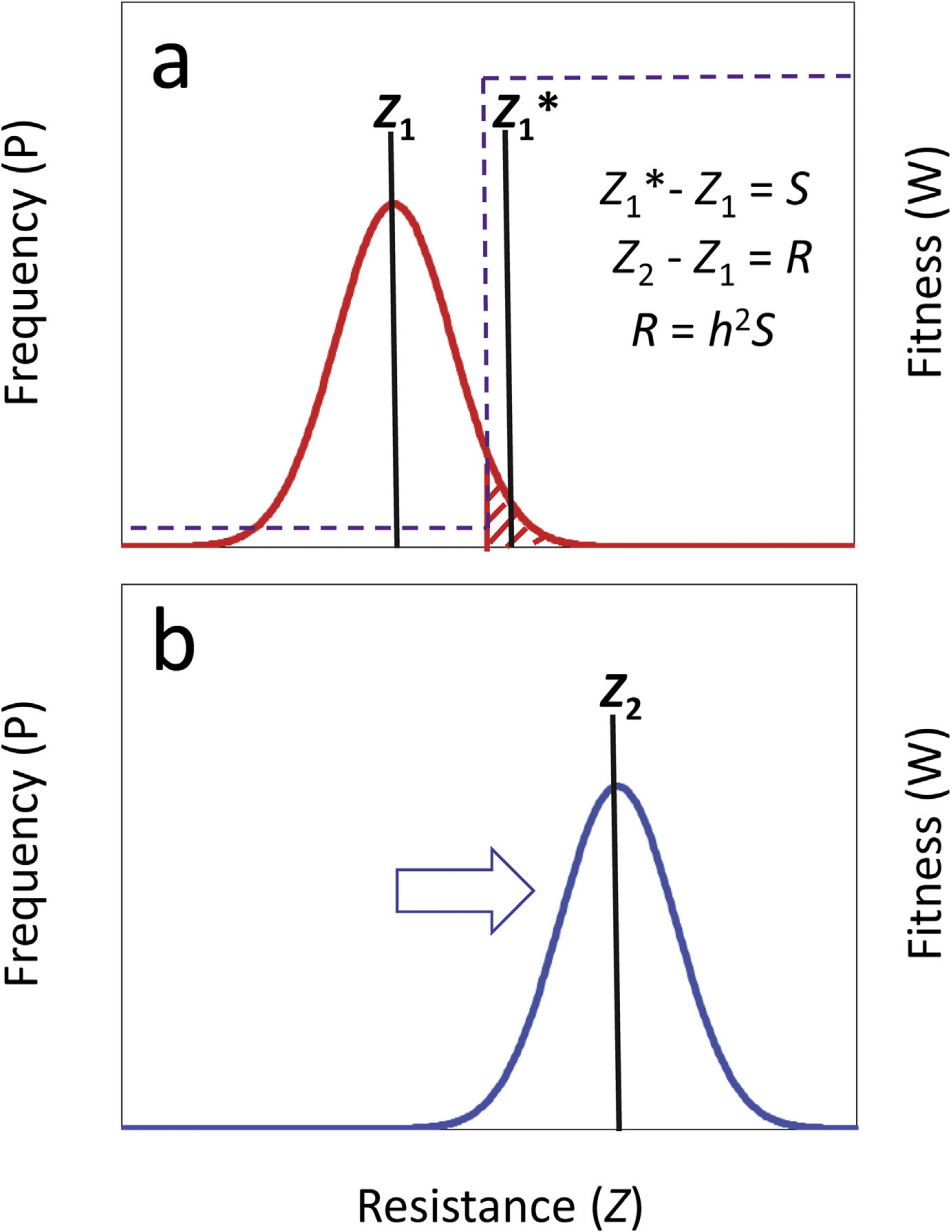
The relationship between the strength of selection, *S*, and the response to selection, *R*, on heritable traits (i.e., those with heritability, *h*^2^>0) is captured by the breeders' equation, *R* = *h*^2^*S*; (a) when a population before selection (red line) experiences truncation selection (dashed purple line), of the form that is likely with the application of pesticides (hatched area = non-responders), *S* equals the difference between the mean of the selected individuals, Z*_1_, and the population mean before selection, *Z*_1_ (*S* = Z*_1_ − *Z*_1_); (b) when a population responds to selection (blue line; direction of response indicated by blue arrow) the response to selection, *R* equals the difference between the mean of the population after selection, *Z*_2_, and the population mean before selection, *Z*_1_ (*R* = *Z*_2_ − *Z*_1_); in each distribution, the average resistance phenotype, *Z*_i_, equals Σ(*z*_*ij*_)/*n*_*ij*_, where *z*_*ij*_ equals the resistance phenotype of each *j*-th individual within the *i*-th resistance trait distribution (1 or 2), and *n*_*ij*_, = the total number of *j*-individuals within the *i*-th resistance trait distribution; in most applications the differences between population means are divided by the standard deviation of the initial population, not shown here because these distributions are assumed to be unit normal; although this approach can be used to estimate selection intensity associated with pesticide application, execution usually requires a controlled laboratory environment (Becker, 1985).

The evolutionary mechanism underlying evolved resistance is easily understood. When pest populations are exposed to a pesticide, only the fraction of individuals who are non-responsive, or who require higher than average concentrations to respond (i.e., “non-responders”), remain within the population. When non-responders leave progeny, they pass genetic factors responsible for their non-responsiveness to offspring, allowing the population fraction eliminated by the pesticide to be replaced by individuals who are resistant to its later application (Fig. 1). Repetition of this sequence with higher treatment concentrations leads to increasingly resistant populations of pests (i.e., higher proportions of non-responders within the population), a process that threatens to make most pesticides obsolete (May, 1985; Ishizuka et al., 2008). Evolved resistance is now recognized as one of the most significant problems of modern times (Garrett, 1994; Palumbi, 2001; Davies and Davies, 2010; Frieri et al., 2016; Gould et al., 2018).

Three primary solutions have been proposed to mitigate selection favoring the evolution of pesticide resistance (other less common approaches are cited below): (1) *Withholding treatment altogether*, (2) *Selectively applying treatment* and (3) *Applying treatments in combination*. The first approach rests on the assumption that pest populations cannot evolve resistance to chemicals they do not experience (Garrett, 1994, microbes, arthropods, rodents; Adegbola et al., 2012, rats; Zhu et al., 2017, insects). However, implementation of this method defeats the purpose of developing treatments in the first place, and while often recommended, with the rise of increasingly persistent pests, it is seldom employed for long (Palumbi, 2001, viruses, microbes, plants and animals).

The second approach, *selectively applying treatment,* involves targeting treatments to particular pests, and providing specific remedies long enough and in high enough concentrations to eliminate the pest populations entirely (Takahashi et al., 2017, transgenic crops and susceptible arthropods). A variant of this approach includes integrated pest management, in which locally variable application of treatments creates a spatially heterogeneous environment of selection intensities, designed to slow the rate at which resistance evolves (Tabashnik et al., 2003, 2008; lepidopterous insects). Application of this method has been effective in reducing total selection favoring resistance. However, even carefully managed programs tend only to delay the evolution of resistance except in the rare instances in which the pests are indeed extirpated (Wilkinson and Priddle, 2011; e.g., populations of rats removed from some islands; however, the authors suggest that reinvasion is likely).

The third approach, *applying treatments in combination*, is designed to reduce the need to apply high concentrations of any particular treatment and attacks pest vulnerability from multiple directions at once (Hoy, 1998, traditional and transgenic treatments of arthropod pests; Baert et al., 2013, rats). While this approach also seems to delay the evolution of resistance, in all cases known, resistance eventually evolves, often with the added complication that the resulting pests have become resistant to multiple pesticides. This process appears to occur in part because the cellular mechanisms associated with metabolizing different pesticides tend to be genetically correlated (Leslie et al., 2005, rats; Drury et al., 2017, *Tribolium*, applications to all taxa).

An approach for mitigating evolved pesticide resistance that avoids the use of pesticides altogether involves introducing sterilized individuals, usually males, into naturally breeding populations (Knipling, 1959; Knipling and McGuire, 1972; Krasfur, 1998; Klassen and Curtis, 2015). This procedure is designed to decrease or obliterate the fertility of normally breeding females and so control the size of the pest population. However, like the above methods, while initially considered successful in eradicating pest populations, accumulating evidence now suggests that this approach has been less successful than originally claimed (Ant et al., 2012; Perez-Staples et al., 2013; Balestrino et al., 2017; USDA report 2016). One source of difficulty for this methodology appears to have been the evolution of populations that cease to respond to treatment. In particular, females in treated populations appear to evolve the ability to discriminate against treated males, evidently due to intense fertility selection on females favoring individuals who avoid sterile mates, and possibly due to sexual selection enhancing such assortative mating (Hibino and Iwahashi, 1991; Perez-Staples et al., 2013). Techniques for generating sterile males include ionizing radiation (reviews in VanDyck et al., 2005; Balestrino et al., 2017), conditional-lethal genotypes (Ant et al., 2012), and CRISPR-cas9 applications (Borel, 2017; Drury et al., 2017). Additional detail on the approaches described above are available elsewhere (Palumbi, 2001; Tabashnik et al., 2008; Frieri et al., 2016; Takahashi et al., 2017; Harris, 2017; Borel, 2017).

Another management approach used primarily for mammalian pests, involves fertility control (Knipling and McGuire, 1972; Bomford, 1990; Norbury, 2000; Kirkpatrick, 2007). In addition to surgical sterilization, which is used mainly to test specific management hypotheses (Jacob et al., 2006, 2008), two primary methods, immunocontraceptive vaccines and chemical contraceptives, now exist (Norbury, 2000; Magiafoglou et al., 2003; Fagerstone et al., 2010). Although effective for controlling the fertility of larger species (see Cooper and Herbert, 2001; Cooper and Larsen, 2006 on the limitations of this approach), immunocontraceptives require individual injections and are thus impractical for small species in which a large fraction of the population must be treated. For these latter species, chemical contraceptives are more practical because they can be delivered using baits. Chemical contraceptives may interfere with ovulation, fertilization, implantation or ovarian development in females and with spermatogenesis in males (Fagerstone et al., 2010; Dyer and Mayer, 2014). For example, combinations of 4-vinylcyclohexene diepoxide and triptolide have been shown to be highly effective in modifying *Rattus* life history traits. In females, litter number is reduced by increasing the overall concentration in each dose; litter size is reduced by varying dosage frequency; in males, even greater sensitivity to dosage exists and may lead to lifelong sterility in some individuals (Mayer et al., 2002, 2004; Dyer et al., 2013; Dyer and Mayer, 2014; Pyzyna et al., 2014). Note that while chemically induced sterility can often be induced in both sexes, this is usually not the means by which males are sterilized in the sterile-male treatments of pest populations described above (Knipling, 1959; Knipling and McGuire, 1972; Krasfur, 1998; Klassen and Curtis, 2015).

A large number of chemosterilants exist. Many of these agents have sex-specific effects, most are not species-specific, and nearly all can be difficult to deliver to large populations (Norbury, 2000). The demographic consequences of fertility control are well studied, particularly for rodents (Knipling and McGuire, 1972; Shi et al., 2002), although the results of these studies are mixed. Predicted declines in population size and growth are not always observed (Jacob et al., 2006, 2008). Moreover, while reproductive compensation and other density-dependent effects within the untreated portion of the population have been documented in rabbits (Twigg et al., 2000), mice (Chambers et al., 1999a, Chambers et al., 1999b) and other mammals (Tyndale-Biscoe, 1994; Wolff, 2003), in rats, reproductive compensation and density dependent effects appear to be either seasonal in ways that do not positively influence population growth (Davis et al., 2003), or do not occur consistently within species (Jacob et al., 2006). In general, the genetic basis for immunological responses used to mediate fertility control, as well as the evolutionary effects of selection on resistance to contraceptives are poorly known (reviews in Cooper and Herbert, 2001; Cooper and Larsen, 2006; Magiafoglou et al., 2003; Ransom et al., 2014).

Previous research has shown the impressive potential for population growth possessed by female rats (*Rattus* sp.; Leslie et al., 1952; Knipling and McGuire, 1972; Clark and Price, 1981; Singleton and Petch, 1994; Keiner, 2005). Individual females in most species begin reproducing within 25 days of their birth and may live for three or more years. During their lifetimes females may produce six to nine litters per year with litter sizes ranging between six and 12 pups per litter, although litters as large as 20 are known. Despite aggressive campaigns to cull rat populations, *R. norvegicus* numbers are estimated to exceed 175 million in the US alone, and attempts at population control cost in excess of $20 billion each year (Pimentel et al., 2005). Additional consequences and costs of *Rattus* populations' explosive rates of increase, as well as current methods for rodent control are reviewed in detail elsewhere (Seamark, 2001; Singleton, 2003; Jacob et al., 2006, 2008; Pyzyna et al., 2014).

We have three primary goals in presenting our results. Our first goal is to provide an estimate of the magnitude of selection that can be imposed on pest populations when pesticides, or treatments that induce sterility (sterility inducers) are used for pest population control. Our estimates of the strength of such selection provide a sobering reminder of why pesticide resistance can evolve so quickly and why it has become widespread. Our second goal is to suggest that the selection responsible for the evolution of non-responsiveness to pesticides and sterility-inducers, is similar in its action to sexual selection, and for this reason can be orders of magnitude stronger than that which exists for untreated populations, particularly when treatments have sex-specific effects. Our third goal is to illustrate an approach for delaying, and possibly eliminating, evolved resistance to treatments used for pest population control. Although the principles we describe may apply broadly to the evolution of resistance to all forms of chemical and biological control, our framework compares the strength of selection that exists in two contexts for rats: (1) that which exists for pest treatments that cause sterility or death (pesticides and sterility inducers), and (2) that which exists with pest treatments that reduce fertility (contraceptives).

We present our illustrations in terms of the opportunity for selection (Crow, 1958, 1962; Wade, 1979), which provides a dimensionless, empirical estimate of the maximum strength of selection acting on a particular population (Wade, 1979; Shuster and Wade, 2003). Our approach considers the reproductive life history of a hypothetical pest species, biologically similar to the Norway rat, *Rattus norwegicus*, and is designed to provide a comparison of the effectiveness of other pest control measures that kill, sterilize or reduce the fertility of this and similar species (Sridhara and Krishnamurthy, 1992; Singleton and Petch, 1994; Mayer et al., 2002, 2004; Dyer et al., 2013; Dyer and Mayer, 2014; Pyzyna et al., 2014). Although our example species is a rodent, our framework can apply broadly to a wide range of iteroparous mammals, and also may be appropriate for any non-mammalian pest species for which female reproductive life history parameters can be specified. We emphasize that although we consider life history parameters in our simulations, we have deferred the specific examination of population growth for a future analysis (Shuster et al. in prep). Here, we have focused instead on identifying the strength of selection that is likely responsible for driving the evolution of resistance to pest control treatments.

Our overall conclusion is that mitigating the intense selection that is responsible for the evolution of pesticide resistance can be accomplished by changing the usual goal of pest control treatments. That is, instead of attempting, and ultimately failing, to destroy or sterilize an entire pest population, we advocate the use of contraceptive treatments that primarily act to reduce these species' rates of reproduction, and that leave as much of the pre-treatment population as possible intact. We show that this approach dramatically reduces the opportunity for selection favoring the evolution of resistance and may thereby permit the safe and long-term application of pest control measures. Although we did not model the population growth of pest populations in this paper, following the recommendations of other authors (Leslie et al., 1952; Cole, 1954; Knipling, 1959; Knipling and McGuire, 1972), we assert that when it is possible to reduce the explosive reproductive rate that characterizes most pest populations, more natural regulatory mechanisms are likely to control their population growth. Our results could have significant implications for the application of pesticides, now and into the future.

## 2 Materials and methods

### 2.1 The opportunity for selection

**Fig. 2:**
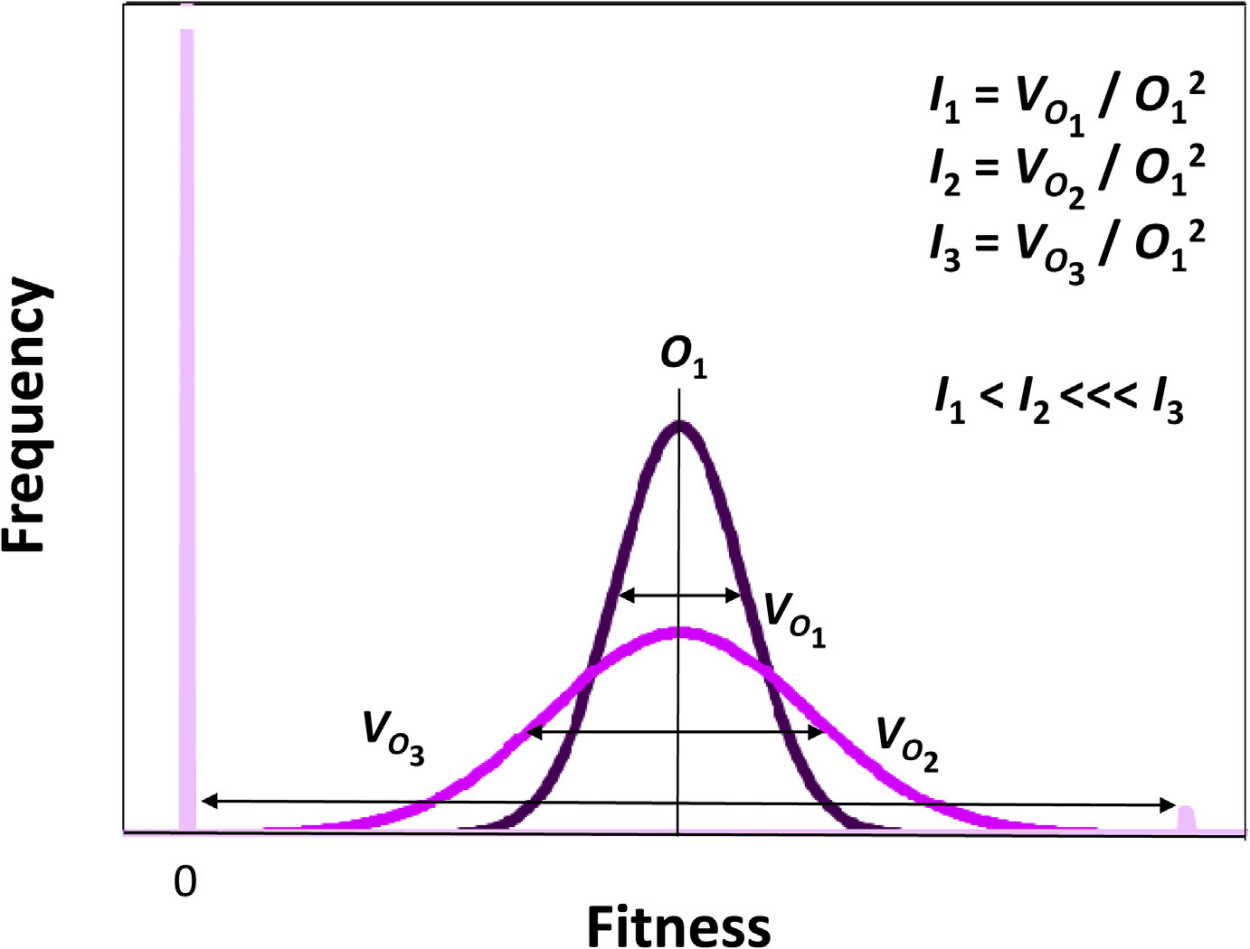
Measuring the opportunity for selection, *I*_*i*_, requires estimates, for the *i*-th fitness distribution (1, 2, 3), of the mean fitness, *O*_*i*_ and variance in fitness, *V*_*Oi*_, usually measured in offspring numbers, where *O*_*i*_ = Σ(*o*_*ij*_)/*n*_*ij*_, and *V*_*Oi*_ = Σ(*o*_*ij*_^*2*^)/*n*_*ij*_, – *O*_*i*_^2^, and where *o*_*ij*_ equals the number of offspring produced by the *j*-th adult within the *i*-th fitness distribution, and *n*_*ij*_, equals the total number of *j*-adults within the *i*-th fitness distribution; because the variance in fitness is proportional to the strength of selection, when average fitness is constant, the larger the variance in fitness variance, the greater the opportunity for selection; when a large number of individuals have zero fitness (e.g., for *I*_3_) as is true in sexual selection and with the application of pesticides and sterility inducers, the magnitude of opportunity for selection can become extreme.

Crow (1958, 1962) observed that the variance in absolute fitness, *V*_*W*_, divided by the squared average fitness, *W*^2^, equals the variance in relative fitness, or *V*_*W*_/*W*^2^ = *V*_*w*_. Crow called this value, the “opportunity for selection,” noting that this parameter measures “total selection intensity.” The opportunity for selection, *I* (= *V*_*W*_/*W*^2^ = *V*_*w*_), has proven useful for providing a dimensionless, empirical estimate of the maximum strength of selection acting on a particular population (Wade, 1979; Arnold and Wade, 1984; Shuster and Wade, 2003; Fig. 2). The opportunity for selection places an upper boundary not only on the change in mean fitness due to selection but also on the change in the standardized mean of every other trait, allowing inferences to be drawn about how selection may act on traits that are difficult or impossible to measure (Krakauer et al., 2011; Moorad and Wade, 2013). The opportunity for selection also has the added advantage of accounting for the “invisible fraction” of the population that may be missing from population samples because of the action of selection itself (Moorad and Wade, 2013; Prather and Shuster, 2016). Methods for estimating selection that emphasize measurements of the covariance between particular phenotypes and relative fitness (Jones, 2009; Klug et al., 2010; Kokko et al., 2012) are unable to incorporate this information as effectively (Krakauer et al., 2011; Moorad and Wade, 2013). Our methods for estimating mean and variance in offspring numbers, and the opportunity for selection on female and male rats are detailed in Appendices A–H.

### 2.2 Simulations of rat reproduction – the effects of litter number and litter size

**Fig. 3:**
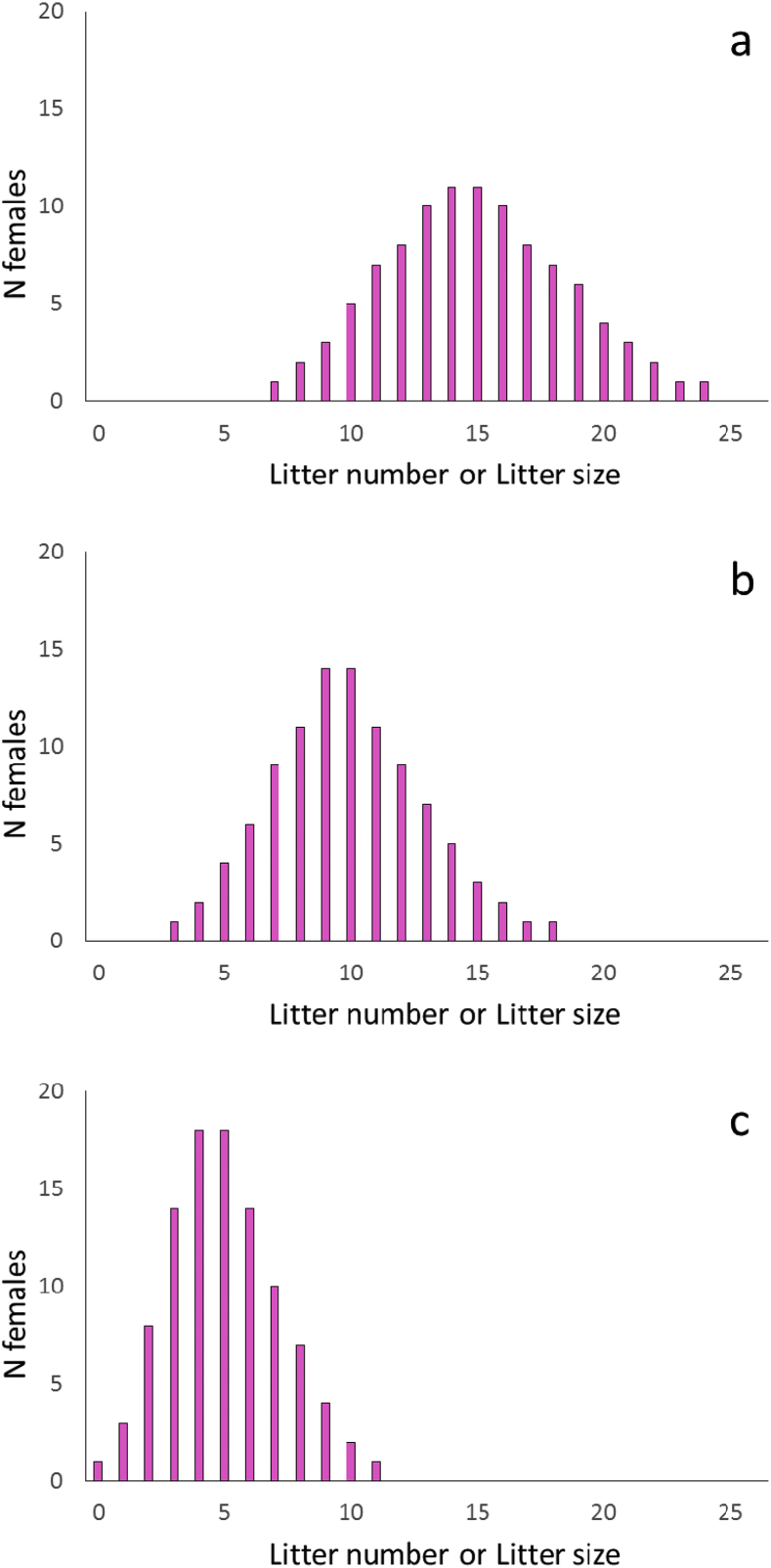
The distribution of litter sizes for females reproducing with an average litter number, *J*, and variance in litter number, *V*_*J*_; or, the distribution of litter numbers for females reproducing with an average number of offspring per litter, *K* and the variance in the number of offspring per litter, *V*_*K*_; *J* and *V*_*J*_ or *K* and *V*_*K*_ are equal to (a) 15, (b) 10 and (c) 5 litters per female or offspring per litter per female, respectively.

We began by generating a normal distribution for the number of litters produced by 100 hypothetical female rats, reproducing with an average litter number, *J*, and a variance in litter number, *V*_*J*_, equal to 15 litters (Appendix A; Fig. 3a–c). We next generated a normal distribution of 100 hypothetical female rats reproducing with an average litter size, *K*, and a variance in litter size, *V*_*K*_, equal to 15 offspring per litter (Appendix B; Fig. 3a–c). Although these simulated distributions of litter sizes and litter numbers were designed to be normally distributed, we made no assumptions that past or ongoing selection were responsible for shaping these trait distributions. We also made no corrections to our distribution to account for the fact that in natural populations, some fraction of females would fail to reproduce by chance alone. We consider the effect of including such assumptions in section 4 below. We performed similar procedures to generate normal distributions of 100 female litter numbers with a mean (*J*) and variance (*V*_*J*_) equal to 10 and 5 litters per female respectively (Appendices A, B). Note that our results are reported with values of *J* and *K* decreasing as 15, 10, 5 to show the effect reductions in litter size and litter number due to the contraceptive treatments described below. We present this sequence in reverse order (5, 10, 15) in tables and in other figures to facilitate graphical presentation.

### 2.3 Quantifying the mean and variance in female offspring numbers

**Fig. 4:**
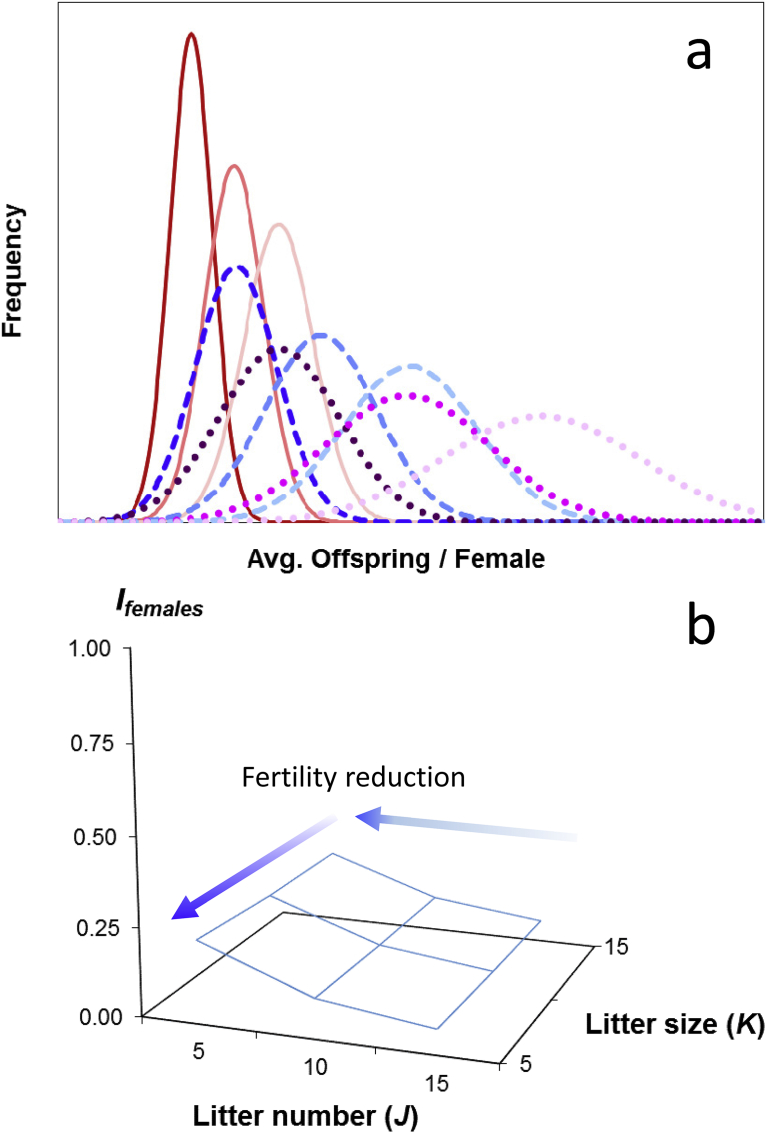
The effects of variation in the average and variance in litter number (*J*) and in the average and variance in litter size (*K*) on the mean and variance in female offspring numbers, *O*_*females(JK)*_, and *V*_*Ofemales(JK)*_, and on opportunity for selection on females, *I*_*females(JK)*_; (a) Distributions of the average, *O*_*females(JK)*_, and the variance, *V*_*Ofemales(JK)*_, in the number of offspring produced by female rats (*R. norvegicus*), arising from variation in two female life history traits, litter number, *J*, and litter size, *K*; red curves: *J* = *V*_*J*_ = 5; *K* =*V*_*K*_ = 5, 10, or 15; blue curves: *J* = *V*_*J*_ = 10, *K* = *V*_*K*_ = 5, 10, or 15; purple curves: *J* = *V*_*J*_ = 15, *K* = *V*_*K*_ = 5, 10, or 15; note that these distributions are shown as continuous curves but in simulations represented integer values for *O*_*females(JK)*_ (see Appendix C); (b) the surface of values for *I*_*females(JK*)_ generated from the values of *O*_*females(JK)*_, and *V*_*Ofemales(JK)*_ from Fig. 2a.; the effect of litter number is slightly more pronounced than litter size on the opportunity for selection on females; specifically, smaller litter numbers cause an increase in the opportunity for selection on females at this scale of female fecundity; however, note that *I*_*females(JK*)_ in all cases is less than 0.25.

By combining the variation in litter number and the variation in litter size from our distributions of these life history parameters (Appendix C; Fig. 3a–c), we next estimated the average, *O*_*females(JK)*_, and variance, *V*_*Ofemales(JK)*_, in the number of offspring produced by females, where *O*_*females(JK)*_ equaled the average number of offspring produced by females within the *J*-th distribution of litter numbers and the *K*-th distribution of litter sizes, and *V*_*Ofemales(JK)*_ equaled the total variance in offspring numbers for females within the *J*-th distribution of litter numbers and the *K*-th distribution of litter sizes (Fig. 4a). Litter number and litter size are likely to be genetically correlated in many rodent species (Cheverud, 1988). Depending on the strength of this correlation in *Rattus*, our combination of the variation in these traits could generate increases in the total variance in offspring numbers as well as in the opportunity for selection on *O*_*females(JK)*_ that are more extreme than we report here. We did not specifically include this effect in our simulations. We assumed that male influences on female offspring numbers were negligible and unless specifically described (see section 2.7), we made no assumptions about whether females had mated once or more than once.

### 2.4 The opportunity for selection on female life history traits

We next estimated the opportunity for selection on females for each of the above distributions by dividing the total variance in offspring numbers, *V*_*Ofemales(JK)*_, by the squared average offspring number, *O*_*females(JK)*_, or,

(1) *I*_*females(JK)*_ = *V*_*Ofemales(JK)*_/(*O*_*females(JK)*_)^2^,

where *I*_*females(JK)*_, equaled the opportunity for selection on females within the *J*-th distribution of litter numbers and the *K*-th distribution of litter sizes, and where *V*_*Ofemales(JK)*_ and *O*_*females(JK)*_ were defined as described above (Table 1; Appendices A–C). To visualize the combined effects of variation in litter number and litter size on the opportunity for selection on females, we generated a surface of values for *I*_*females(JK)*_ by plotting the values for litter number and litter size as independent variables (Figs. 2 and 4b).

**Table 1:** The combined effects of variation in the mean and variance in litter number and litter size on the mean (*O*_*females(JK)*_) and variance (*V*_*Ofemales(JK)*_) in offspring numbers and the opportunity for selection on females (*I*_*females*_); the first row of each cell contains the mean and the variance (in parentheses); the second row contains *I*.

Litter size (*K*)	Litter number (*J*)		
	5	10	15
5	25.0 (146.5)	49.3 (275.8)	74.6 (395.1)
	0.234	0.113	0.071
10	50.5 (536.0)	98.6 (1004.6)	149.2 (1431.2)
	0.214	0.103	0.064
15	75.0 (1168.5)	147.9 (2186.4)	224.0 (3108.3)
	0.208	0.100	0.062

### 2.5 The effect of non-responders on rat reproduction

**Fig. 5:**
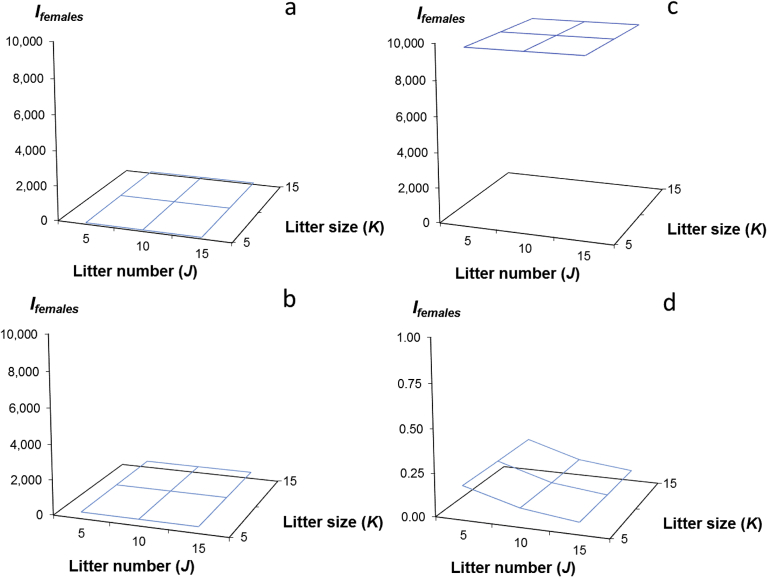
The effect of varying the proportions of responders and non-responders within populations of 100 female rats on the opportunity for selection on females, *I*_*females(JK)*_; here, *I*_*females(JK)*_ is represented as a surface generated by variation in litter number (*J*) and litter size (*K*); in simulations a–c, responders were eliminated from the population entirely by treatment with pesticide or sterility inducer with the following intensities: (a) 90% responders and 10% non-responders; (b) 95% responders and 5% non-responders; (c) 99% responders and 1% non-responders; in simulation (d) 1% non-responders were embedded within populations of 99% responder female rats whose fertility was reduced by a contraceptive; note axes values for *I*_*females(JK)*_ in (a–c) range from 0 to 10,000; for (d) *I*_*females(JK)*_ ranges from 0 to 1.0.

We next examined the effect that most pest control measures have on rat reproduction; specifically, procedures designed to kill or sterilize individuals, and thus that severely reduce the overall size of the reproducing population of pests. As mentioned above, this procedure is seldom completely effective in eliminating all individuals and thereby allows reproduction by the few remaining resistant individuals (Fig. 1). To simulate the intense selection caused by pest treatments, we created three female fitness distributions that differed in their proportions of responders and non-responders (responders:non-responders equaled 90%:10%, 95%:5%, and 99%:1%, N = 100; Appendix D). We estimated the mean, *O*_*females(JK)*_, and variance, *V*_*Ofemales(JK)*_, in offspring numbers produced by females with each life history distribution (*J* = *K*=*V*_*J*_ = *V*_*K*_ = 15, 10, 5; Appendix C), and we estimated the opportunity for selection on reproducing females, *I*_*females(JK)*_, as described above. We then plotted these values as surfaces for each of the three distributions of responders and non-responders (Fig. 5a–c; Tables 1a–c are reported in Appendix D). Note that the variation in the proportions of responders and non-responders we established is combined with reduction in female fecundity, providing a means for examining the effects of the simultaneous application of a pesticide or sterility inducer, with a contraceptive.

### 2.6 The effect of embedding non-responders within populations of responders

We next examined the isolated effect of contraceptives that modify the life history traits of females so that responders remain within the population and are still capable of reproduction, but do so with reduced fertility. As has been shown in previous studies, reduced fertility in female rats can be accomplished using contraceptives by reducing the number of litters or the number of offspring per litter or both (Mayer et al., 2002, 2004; Dyer et al., 2013; Dyer and Mayer, 2014). To simulate this process we modified the distributions of responders and non-responders so that 99% of females reproduced with mean and variance in litter number, *J* and *V*_*J*_, and mean and variance in litter size, *K* and *V*_*K*_, equaling 15, 10, and 5, respectively (Appendices A–D). Then, within each of these distributions, we embedded a single, non-responding female whose litter number and litter size (*JK*) each equaled 15 (i.e., 1/100 females was non-responsive to the treatment), thereby considering the effect of this non-responsiveness within a population of other females whose fertility was *reduced* rather than eliminated entirely. Note that in this simulation, because the non-responding female had *J* = *K* = 15, the fertility distribution of responding females with *J* = *K* = 15 was unmodified relative to the above simulation. We estimated the average and variance in offspring numbers produced by females within each of the now four female fitness distributions (see Appendix D), and we estimated the opportunity for selection on reproducing females as described above (Table 1d, Appendix D). We then plotted these values as a surface as described above (Fig. 5d).

### 2.7 The sex-specific effect of pesticides, sterility-inducers and contraceptives

The above simulations focused only on female reproduction, as is typical of most life history analyses (Ricklefs, 2006). However, as mentioned above, in rats, the sexes may differ in their sensitivity to chemical treatments (Mayer et al., 2002, 2004; Dyer et al., 2013; Dyer and Mayer, 2014; Pyzyna et al., 2014). Moreover, the relationship between mate numbers and offspring numbers differentially influences the total fertility of males and females (Wade, 1979). Sexual selection occurs when certain individuals within one sex produce offspring at the expense of other individuals of the same sex (Darwin, 1871). Whenever a large fraction of one sex fails to reproduce, a sex difference in the opportunity for selection appears (Shuster and Wade, 2003; Wade and Shuster, 2004). Pesticides and sterility inducers produce a similar effect in part because they prevent a large fraction of the treated population from reproducing. Moreover, this effect is enhanced when treatment affects one sex more than the other; and as with sexual selection, the more effective a pesticide or sterility inducer is, the smaller the proportion of individuals is that contributes to the next generation (Figs. 1 and 2). To visualize the sex-specific effect of pesticides, sterility inducers and contraceptives on rat reproduction, and in turn, on the opportunity for selection favoring non-responsiveness to such treatments, we next considered the separate contributions by female and male rats to the total variance in offspring numbers. In terms of the above simulations, for a given treatment, if a larger fraction of one or the other sex failed to reproduce, a sex difference in the opportunity for selection would arise, similar to the action of sexual selection (Wade and Shuster, 2004, 2005).

Wade (1979; see also Fisher, 1958; Shuster and Wade, 2003; Wade and Shuster, 2004; Shuster, 2007; Prather and Shuster, 2016) showed that the mean and variance in male fitness, evaluated in terms of offspring numbers, can be expressed in terms of the mean and variance in female offspring numbers. Because each offspring has a mother and a father (Fisher, 1958; Wade, 1979; Queller, 1987), the average fitnesses of males and females are equivalent and are linked through the sex ratio, *R* (= *N*_*females*_/*N*_*males*_), where *O*_*males*_ = *R O*_*females*_. Note that in addition to describing the sex ratio, *R* equals the average number of mates per male (Shuster and Wade, 2003). When males mate with more than one female, the mean and variance in the fitness of males in each mating class equals the mean and variance in female fitness, multiplied by the number of mates that the males in each mating class obtain. The consequence of this relationship is that, while the average fitnesses of males and females must be equivalent, the variance in male offspring numbers can far exceed the variance in female offspring numbers. Moreover, like the total variance in female offspring numbers, the total variance in male offspring numbers can be partitioned into two components; the variance in fitness within the classes of males who mate successfully, and the variance of the average fitness between the classes of males who mate successfully and those who do not (Wade, 1979; Shuster and Wade, 2003).

Using this approach and the estimates of the mean and variance in female offspring numbers defined in Appendix C, we evaluated the total variance in male offspring numbers for each *JK*-th distribution of female fertility. As mentioned, certain rat contraceptives are known to reduce or eliminate male fertility disproportionately to that of females (Mayer et al., 2002, 2004; Dyer et al., 2013; Dyer and Mayer, 2014; Pyzyna et al., 2014). While it is possible that such treatments may cause all males in a population to become sterile, it is more likely that some fraction of the male population will consist of non-responders, i.e., males who retain their fertility despite experiencing a treatment designed to sterilize males (Pérez-Staples et al., 2013). The smaller the fraction of non-responding males, the larger the fraction of males who do not contribute to the next generation at all, and similar to the action of sexual selection, the stronger the opportunity for selection on males, and the larger the sex difference in the opportunity for selection will be (Shuster and Wade, 2003). Moreover, because in this case, only non-responder males will contribute to the next generation, the opportunity for selection on such a male population represents the opportunity for selection favoring any trait possessed by the favored individuals. In this case, the favored trait is non-responsiveness to the pesticide or sterility-inducing treatment (Shuster and Wade, 2003; Krakauer et al., 2011).

To show the potential magnitude of this effect, we replicated the four treatment scenarios identified for females in sections 2.5 and 2.6 above (Appendix D) with the following exceptions: We removed the 95:05 responder:non-responder treatment, because it was redundant with other treatments in this simulation, and substituted a treatment in which: (1) the treatment effects were presumed to be too subtle to influence females; thus this population consisted of 100% non-responder females, or stated differently, this treatment created a condition in which selection on resistance through females was absent. The other treatment scenarios were: (2) 90% of females were responsive to treatment, (3) 99% of females were responsive to treatment, and (4), 1% non-responsive females were embedded within a population of 99% responders, whose fertility was reduced rather than eliminated by exposure to treatment with a contraceptive (c.f., Appendix D).

**Fig. 6:**
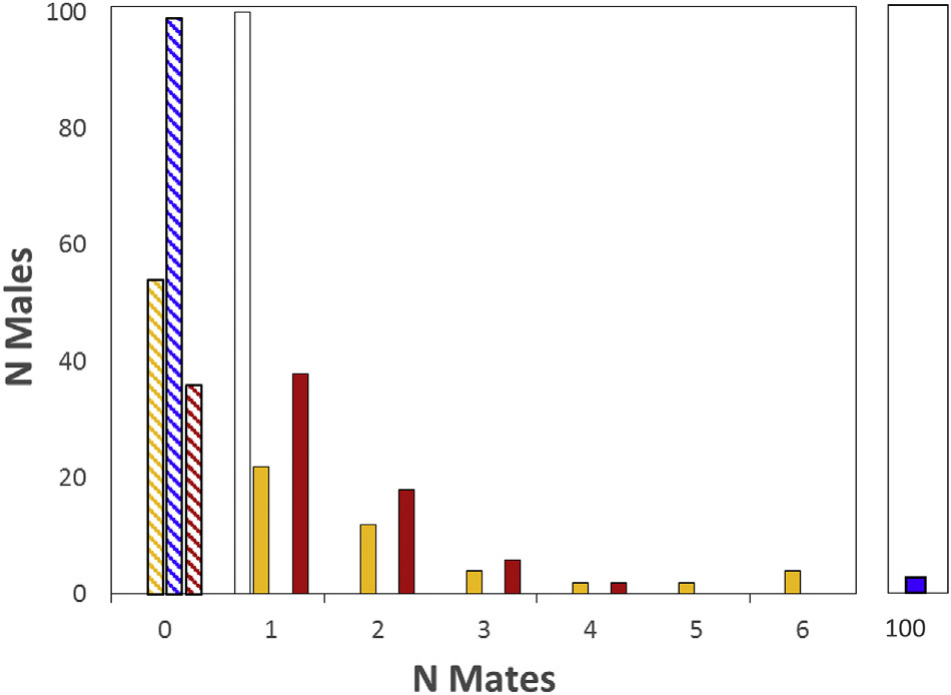
Mating systems that simulate the sex-specific effect on the opportunity for selection on male non-responders to pesticides, sterility-inducers and contraceptives; hatched bars indicate the fraction of the male population that fails to produce offspring, solid bars represent non-responders who are successful in producing offspring; (white; GM; 100% non-responders) genetic monandry, in which males are presumed to mate only once; (red; RM; 64% non-responders) random mating, in which males are presumed to mate randomly with females; (gold; SSM; 46% non-responders) moderate sexual selection, in which the mating success of males is moderately skewed, and (blue; SSE; 1% non-responders) extreme sexual selection, in which the mating success of males is maximally skewed. Note that in RM (random mating), 36% of the males fail to breed due to chance alone, simultaneously illustrating the consequence of the treatment affecting males randomly within the population, as well as the effect that chance has on male mating success.

We next identified the mean and variance in female offspring numbers, *O*_*females(JK)*_, *V*_*Ofemales(JK)*_, generated by the three female fitness distributions obtained when the values of *J* and *K* were made equivalent, as in our focal distributions of female fitness (*J* = *K* = *V*_*J*_ = *V*_*K*_ = 5, 10, 15; note this order facilitated graphing and tabulation). We then used these fitness distributions to examine the effects on male offspring numbers, of four mating systems that simulated the increasingly intense effects of pesticides or sterility-inducers on the proportion of male responders within a population. The distributions of non-responder males were defined by the following mating systems; (1) genetic monandry (GM; 100% non-responders) in which all males were presumed be non-responsive to treatment, to mate with only one female, and to refuse to mate with non-virgin females; note that because each male in this treatment mates with only one unmated female, females also must mate with only one male; we assumed that all females would successfully mate; (2) random mating (RM; 64% non-responders, 36% responders) in which non-responder males were presumed to mate randomly with females; (3) moderate sexual selection (SSM; 46% non-responders, 54% responders) in which the mating success of non-responder males was moderately skewed, and (4) extreme sexual selection (SSE; 1% non-responders, 99% responders) in which the mating success of responder males was maximally skewed; Appendix E; Fig. 6).

We tabulated our results (Tables 2; Tables 2a–b, Appendix E) as follows: We used the values of *JK* from Tables 1a–1d (Appendix D), in which *J* = *V*_*J*_ = *K* = *V*_*K*_ = 5, 10 and 15, as the header entries for the rows which summarized the female fitness distributions. We then summarized the values of the female life history parameters for each of these distributions, including the mean and variance in female offspring numbers, *O*_*females(JK)*_, *V*_*Ofemales(JK)*_, and the opportunity for selection on females, *I*_*females(JK)*_. We then summarized in columns identified for each male mating system (GM, RM, SSM, SSE; Appendix E), the following male life history and mating system parameters: the variance in male offspring numbers, *V*_*Omales(JK)*_ (evaluated as described in Appendix F), the opportunity for selection on males, *I*_*males(JK)*_ (evaluated as described in Appendix F), the ratio, *I*_*males(JK)*_/*I*_*females(JK)*_, as an index of the degree to which a sex difference in the opportunity for selection might be responsible for trait divergence, and *I*_*average(JK)*_, the average opportunity for selection between males and females (evaluated as described in Appendix G).

**Table 2:** The effect of mating system on the opportunity for sexual selection in a rat population with no prior exposure to treatment.

Female fitness distribution	Female parameters			Male parameters	Male mating system			
(*J* = *V*_*J*_ = *K*=*V*_*K*_)	*O*_*females(JK)*_	*V*_*Ofemales(JK)*_	*I*_*females*_		GM	RM	SSM	SSE
5	25.00	146.50	0.234	*V*_*Omales*_	146.50	76.50	1,596.50	62,021.50
				*I*_*males*_	0.234	1.194	2.554	99.200
				*I*_*males*_/*I*_*females*_	1.00	5.10	10.90	423.36
				*I*_*average*_	0.23	0.71	1.39	49.72
10	98.60	1004.60	0.103	*V*_*Omales*_	1,004.60	10,337.70	23,559.50	9,063,478.60
				*I*_*males*_	0.103	1.063	2.423	99.103
				*I*_*males*_/*I*_*females*_	1.00	10.29	23.45	959.07
				*I*_*average*_	0.10	0.58	1.26	49.60
15	224.00	3108.30	0.062	*V*_*Omales*_	3,108.30	1,277.26	119,516.60	4,970,532.30
				*I*_*males*_	0.062	1.022	2.382	99.062
				*I*_*males*_/*I*_*females*_	1.00	16.50	38.45	1,599.12
				*I*_*average*_	0.06	0.54	1.22	49.56

GM = genetic monogamy; RM = random mating; SSM = moderate sexual selection; SSE = extreme sexual selection (details provided in text).

**Fig. 7:**
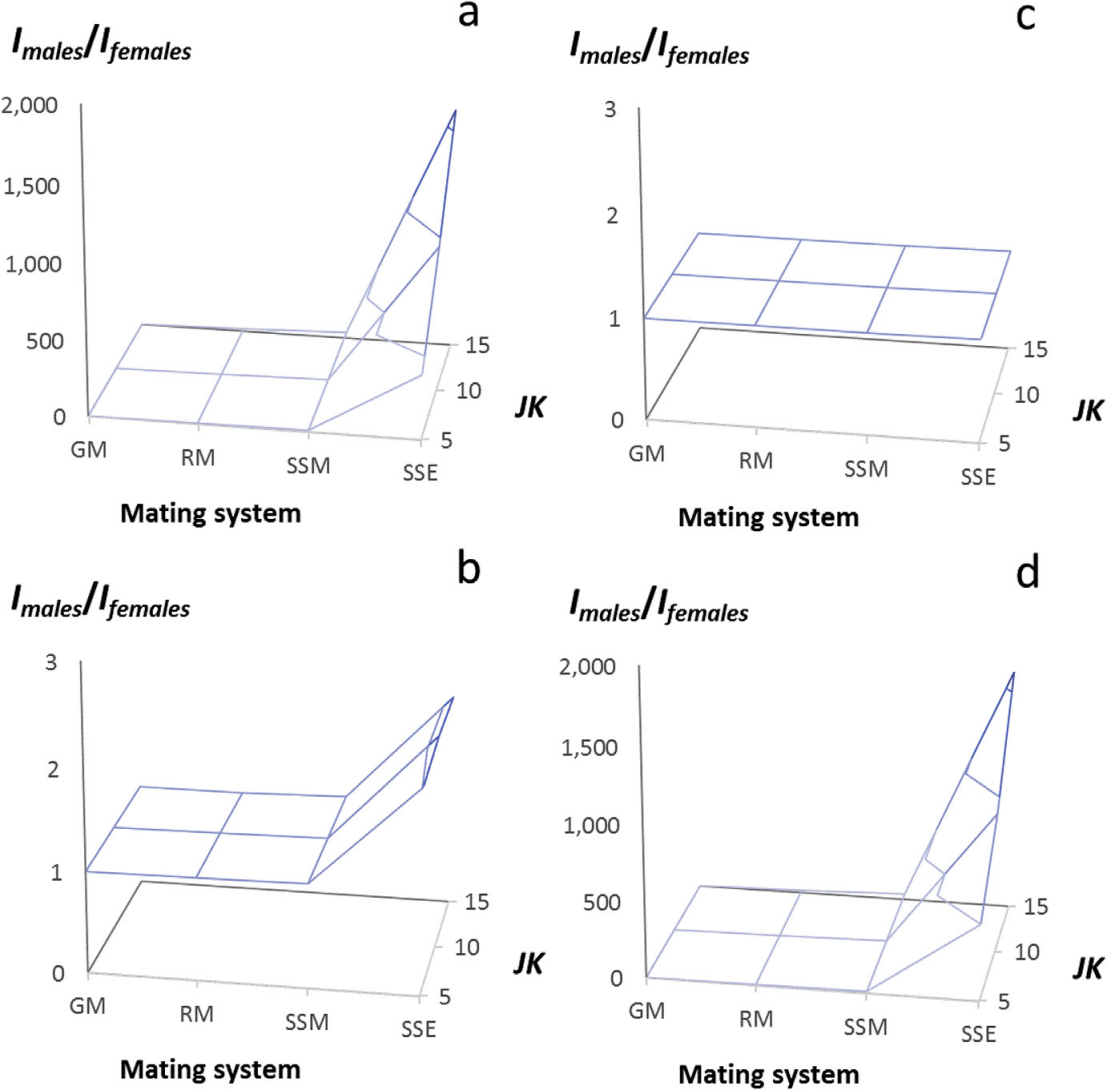
The magnitude of the sex difference in the opportunity for selection, *I*_*males(JK)*_/*I*_*females(JK)*_, resulting from varying the proportions of responders and non-responders within populations of 100 female rats, and mating these females to 100 males whose proportions of responders and non-responders were determined the mating system; female proportions of responders: non-responders were (a) 0.0:1.0; (b) 0.90:0.10; (c) 0.99:0.01 (d) embedding 0.01 non-responders within populations of 0.99 responder female rats whose fertility was reduced by a contraceptive; male proportions of responders: non-responders were: GM (genetic monandry, 0.0:1.0), RM (random mating, 0.36:0.64), SSM (moderate sexual selection, 0.54:0.46), SSE (extreme sexual selection, 0.99:0.01); *I*_*males(JK)*_/*I*_*females(JK)*_ is represented as a surface generated by variation in female fecundity resulting from variation in litter number (*J*) and litter size (*K*); note axes values for *I*_*males(JK)*_/*I*_*females(JK)*_ in (a, d) range from 0 to 2,000; for (d) *I*_*females(JK)*_ ranges from 0 to 3.0.

**Fig. 8:**
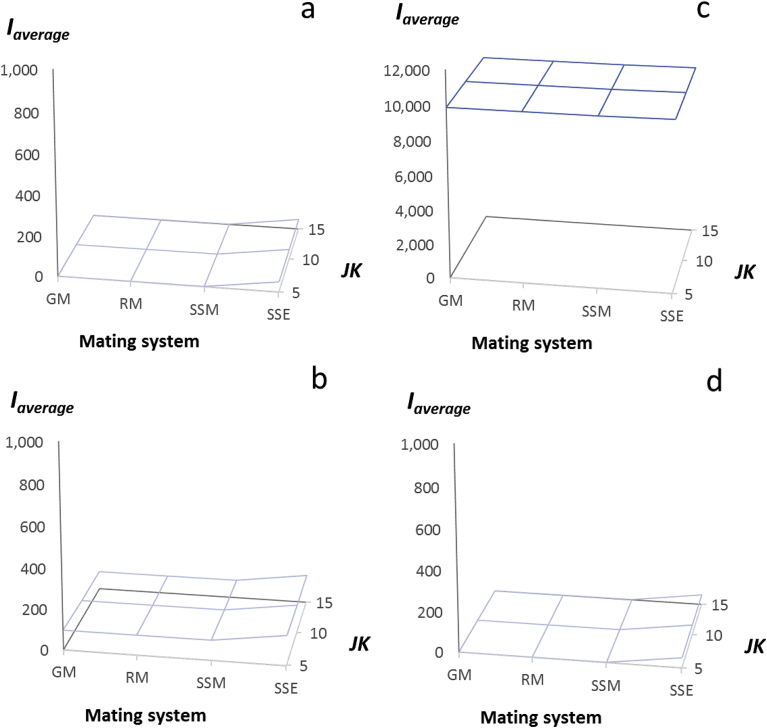
The magnitude of the average opportunity for selection between the sexes, *I*_*average(JK)*_, resulting from varying the proportions of responders and non-responders within populations of 100 female rats, and mating these females to 100 males whose proportions of responders and non-responders were determined the mating system (GM, RM, SSM, SSE); female proportions of responders: non-responders were (a) 0.0:1.0; (b) 0.90:0.10; (c) 0.99:0.01 (d) embedding 0.01 non-responders within populations of 0.99 responder female rats whose fertility was reduced by a contraceptive; male proportions of responders: non-responders were: GM (genetic monandry, 0.0:1.0), RM (random mating, 0.36:0.64), SSM (moderate sexual selection, 0.54:0.46), SSE (extreme sexual selection, 0.99:0.01); *I*_*average(JK)*_ is represented as a surface generated by variation in female fecundity resulting from variation in litter number (*J*) and litter size (*K*); note axes values for *I*_*average(JK)*_ in (a, b, d) range from 0 to 1,000; for (c) *I*_*average(JK)*_ ranges from 0 to 12,000.

To provide additional clarity, for each of the four treatment scenarios in which the proportions of female responders and non-responders were varied (see 2.5 and 2.6 above; Appendix D), we plotted as surfaces, the ratio, *I*_*males(JK)*_/*I*_*females(JK)*_, resulting from the three female fitness distributions (*JK* = 5, 10, 15) and the four male mating systems, which served to represent increasing intensities of pest control treatment (Fig. 6), as well as the average opportunity for selection for both sexes, *I*_*average(JK)*_ resulting from the three female fitness distributions (*JK* = 5, 10, 15) and the four male mating systems (Fig. 7). The ratio, *I*_*males(JK)*_/*I*_*females(JK)*_), showed how much stronger selection was in one sex compared to the other. The average opportunity for selection, *I*_*average(JK)*_, showed the aggregate effect of selection acting through males and females, as a consequence of pesticides, sterility inducers or contraceptives (Fig. 8).

### 2.8 Assortative mating and the evolution of resistance to pesticides, sterility-inducers and contraceptives

Lastly, we considered the effect of assortative mating on the opportunity for selection in males, wherein females preferred to mate with males bearing certain traits, and preferred not to mate with males lacking these traits (Appendix H). We considered this condition likely to affect the opportunity for selection favoring resistance to pesticides, sterility-inducers and contraceptives, if non-responding females developed a tendency to mate preferentially with non-responding males (positive assortative mating). If female mate preference is based on the degree to which males express the preferred trait, and if these two traits are heritable, a positive genetic correlation is expected to arise, possibly leading to “run-away” exaggeration of both the male trait and the female preference for it (Fisher, 1958; Lande, 1981; Kirkpatrick, 1982; Shuster and Wade, 2003).

Positive assortative mating associated with non-responsiveness to a pesticide, sterility-inducer or contraceptive, could accelerate the evolution of treatment resistance (Drury et al., 2017). Such conditions are known to influence attempts at biological control of insect pests in which sterile males were introduced to populations but females developed mate preferences for non-sterile (i.e., non-responding) males (Hibino and Iwahashi, 1991; Pérez-Staples et al., 2013; Drury et al., 2017). Negative assortative mating, in which non-responding females prefer to mate with responding males, or in which responding females prefer to mate with non-responding males, is expected to decelerate the evolution of pesticide resistance because it creates negative genetic correlations between the male traits and female preferences for them (Shuster and Wade, 2003; Hedrick et al., 2016). Selection favoring male responders is weakened if such males tend to mate primarily with non-responder females, and similarly, selection favoring non-responder females is weakened if such females mate primarily with responder males.

We considered four treatment scenarios with conditions identical to those described in section 2.7 above: (1) 100% of females were non-responders; as mentioned this provided a condition in which selection on resistance through females was absent; (2) 90% of females were responsive to treatment, (3) 99% of females were responsive to treatment, and (4), 1% non-responsive females were embedded within a population of 99% responders, whose fertility was reduced rather than eliminated by exposure to treatment with a contraceptive (c.f., Appendix D). Within these conditions, we varied the degree to which females mated randomly with males (*s* = 0) or whether females preferred to mate with particular males (*s* = 1 to 5). As described above we first generated distributions for the mean and variance in female offspring numbers as shown in Table 1, 1a–d, Appendix D.

**Fig. 9:**
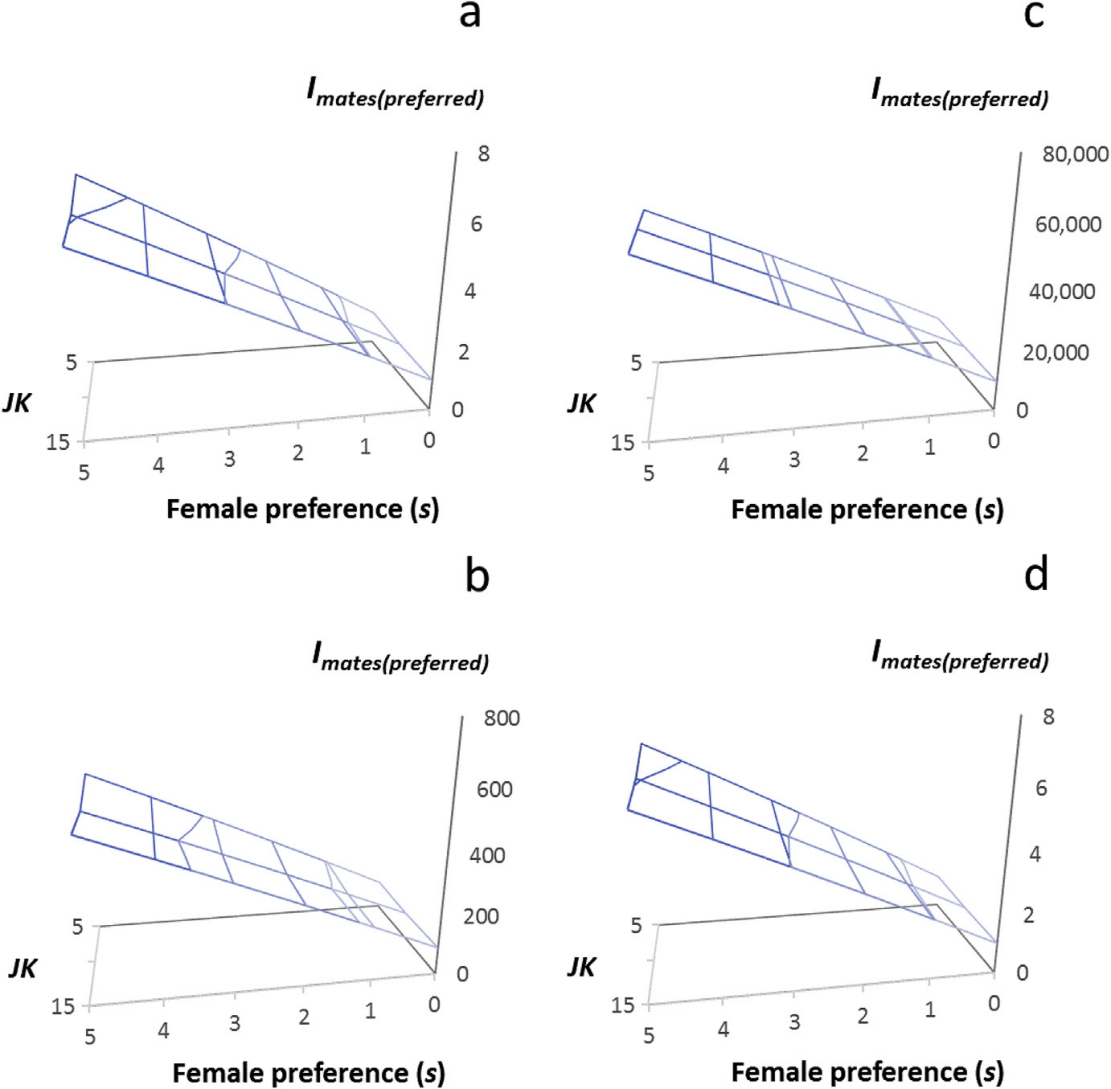
The magnitude of the opportunity for selection, *I*_*mates(preferred)*_, resulting from female mate preferences for males with particular traits; *I*_*mates(preferred)*_ is represented as a surface generated representing by variation in female fecundity resulting from combined variation in litter number and litter size (*JK=* 5, 10, 15), and from variation in the intensity of female mate preferences; *s* = 0 indicates random mating; *s* = 1–5 indicates increasing intensity of female mate preferences; female proportions of responders: non-responders were (a) 0.0:1.0; (b) 0.90:0.10; (c) 0.99:0.01 (d) embedding 0.01 non-responders within populations of 0.99 responder female rats whose fertility was reduced by a contraceptive; note axes values for *I*_*mates(preferred)*_ in (a, d) range from 0 to 9.0; for (b) *I*_*mates(preferred)*_ ranges from 0 to 800; for (c) *I*_*mates(preferred)*_ ranges from 0 to 80,000.

We next estimated the opportunity for selection on males when females mated at random, *I*_*mates(random)*_, to simulate *s* = 0. We then used Eq. H.2, Appendix H to generate values for *I*_*mates(preferred)*_ by varying the value of s from 1 to 5. To show how a female preference for males resistant to pesticide, sterility inducer or contraceptive would enhance the opportunity for selection on males favoring resistance beyond that expected under random mating, we then plotted for each distribution of responders and non-responders, the resulting values for *I*_*mates(preferred)*_ as a surface dependent on female offspring numbers, indicated by the average litter size and litter number (*JK* = 5, 10, 15), and on the magnitude of the female preference (*s* = 0–5; Fig. 9).

We did not explore the specific effect of negative assortative mating on the opportunity for sexual selection on males favoring male resistance. This pattern of mate preference requires the evolution of a more complicated sequence of behavioral traits and is therefore less likely to arise than positive assortative mating (Shuster and Wade, 2003). However, if negative assortative mating were to arise, it is expected to decrease the opportunity for selection that exists for males, in the absence of female mate preferences, by an amount equal to the magnitude of *I*_*mates(preferred)*_ for a given intensity of the female preference, *s*.

## 3 Results

### 3.1 The distributions of litter number and litter size

We generated three distributions describing the average litter number, *J* and the variance in litter number, *V*_*J*_, for the 100 hypothetical female rats, with *J* = *V*_*J*_ = 15, 10 and 5 litters per female. We also generated three distributions describing the average litter size, *K*, and the variance in litter size, *V*_*K*_ for these same female rats, with *K* = *V*_*K*_ = 15, 10 and 5 offspring per litter per female. Because the distributions describing the mean and variance in litter number and litter size were identical in form to those generated for the mean and variance in litter number, we summarized both life history traits in a single figure (Fig. 3a–c).

### 3.2 The average and variance in offspring numbers and the opportunity for selection on females

Our simulations of the combined effects of variation in mean and variance in the number of offspring produced by females (whose average and variance in litter number, *J* and *V*_*J*_, and whose average and variance in litter size, *K* and *V*_*K*_, equaled 5, 10 and 15), generated the results we expected. As litter number and litter size increased, the average in female offspring numbers, *O*_*females(JK)*_ and variance in female offspring numbers, *V*_*Ofemales(JK)*_ also increased (Fig. 4a; Table 1). Similarly, our estimates of the opportunity for selection on females, *I*_*females(JK)*_, showed that this value decreased with increasing average litter size and litter number, although the rate of decrease in the opportunity for selection was slightly more pronounced with variation in litter number (*J*) than with variation in litter size (*K*; Table 1, Fig. 4b).

### 3.3 The effect of non-responders on female fitness

Our simulations of how variation in the population frequencies of non-responders to pesticides, sterility-inducers and contraceptives, affected the mean and variance in offspring numbers, produced three primary results. First, the existence of a large proportion of individuals that were responsive to pesticide and sterility-inducer treatment (90–99%), and therefore that produced no offspring, decreased the average, and increased the variance in offspring numbers produced by females in our simulations (Tables 1a–1c, Appendix D). This relationship is increasingly well-documented in studies in which the non-reproducing members of the population are included within estimates of the mean and variance in fitness (Shuster and Wade, 2003; Shuster, 2009; Prather and Shuster, 2016). As expected, this effect became more pronounced as the fraction of responders increased (Tables 1a–1c, Appendix D).

Second, within each simulation, estimates of the opportunity for selection on females while slightly different in value, were similar in magnitude, regardless of the values of litter size and litter number (Figs. 4b and 5a–c). This outcome appears to result from the comparatively small ranges of litter size and litter number within this simulation (*J*, *K* = 5, 10, 15). A broader range of life history parameters considered might produce more varied results. However, the life history values considered here seemed appropriate for this pest species (rats), and suggest, consistent with classical ecological theory (Cole, 1954), that the relative influences of litter size and litter number on rat life history in particular, and on life history strategies in general (e.g., semelparity vs. iteroparity) are approximately equivalent. This result also suggests that reducing the litter size and litter number of non-responder females, as might occur if contraceptives were used in combination with the pesticides or the sterility-inducers used to control these hypothetical rat populations, had no recognizable effect on the opportunity for selection favoring non-responsiveness, relative to the effects of selection imposed by the pesticide or sterility inducer.

Third, among our simulations, as the fraction of non-responders to this treatment decreased, and concomitantly, as the fraction of responders to a pesticide or sterility inducer increased, the opportunity for selection on females increased rapidly. Overall, values of *I*_*females(JK)*_ with 10% non-responders were three orders of magnitude greater than values of *I*_*females(JK)*_ in populations untreated with pesticide or sterility inducer (1,500-fold; Fig. 5a–c, Table 1; Table 1a, Appendix D), and values of *I*_*females(JK)*_ with 1% non-responders were two orders of magnitude greater than values of *I*_*females(JK)*_ in populations with 10% non-responders (110-fold; Fig. 5a–c; Tables 1a–c, Appendix D).

This result also indicates that for a pest population with life history characters similar to those considered here, when a pesticide or sterility-inducer eliminates reproduction by 99% of the population, the opportunity for selection favoring non-responsiveness to the pesticide or sterility-inducer can be more than five orders of magnitude stronger (165 thousand-fold) than if selection had occurred by chance alone (Table 1).

### 3.4 The effect of embedding non-responders within populations of responders with reduced fecundity

Embedding 1% non-responders within populations of responders, whose response to treatment was a reduction in fecundity, that is, when treatment consisted of a contraceptive rather than a pesticide or sterility inducer, showed a clear result. Whereas the opportunity for selection favoring resistance increased 165 thousand-fold in a population consisting of 1% non-responders and 99% responders (Fig. 5c; Table 1c, Appendix D), a mixture of 1% non-responders and 99% responders who fertility was reduced by a contraceptive, returned the variation in the mean and variance in fitness and in the opportunity for selection on females, to values approximately equivalent to that for simulated populations in which no treatment was applied (Fig. 4b, Table 1; Fig. 5d; Table 1d, Appendix D). Stated differently, embedding non-responders within a population of responders with reduced fecundity rendered selection favoring non-responsiveness to treatment to a level no greater than would be expected by chance alone.

### 3.5 The sex-specific effect of pesticides, sterility-inducers and contraceptives

We confirmed that sex-differences in responsiveness to a pesticide, sterility inducer or contraceptive, is similar in its action to sexual selection, and therefore will enhance the strength of, as well as complicate the estimation of selection favoring resistance to such treatments. Our first scenario considered a female population that was uniformly non-responsive to pesticide, sterility inducer or contraceptive treatment. We considered this condition equivalent either to a female population with no previous exposure to treatment, or one in which females were less responsive to treatment than males and so were phenotypically “non-responders” (see Table 1). We allowed these females to mate with males who were differentially responsive to treatment, such that only non-responder males were able to produce offspring. We simulated this condition by making decreasing proportions of non-responder males equivalent to mating systems with increasing intensities of sexual selection (GM, RM, SSM, SSE; Fig. 6).

Because 100% of females in this treatment were non-responders to treatment with pesticide or sterility inducer, and under genetic monandry (GM), 100% of males also were non-responders, and males were assumed to mate once and only when a virgin female, this condition amounted to “genetic monogamy.” As expected, this condition returned fitness parameters for males, *V*_*Omales(JK)*_, and *I*_*males(JK)*_, that were identical to these parameters for females in all three female fitness distributions (*J=K* = 5, 10, 15; Table 2). The ratio of *I*_*males(JK)*_/*I*_*females(JK)*_, i.e., our index of the magnitude of the sex difference in the opportunity for selection, also as expected, was equal to 1 (Tables 1 and 2; Fig. 7a). Similarly, the average opportunity for selection between males and females, *I*_*average(JK)*_, the opportunity for selection on females, *I*_*females(JK)*_, and the opportunity for selection on males, *I*_*males(JK)*_, were all equivalent in value (=0.234, Table 2; Fig. 8a).

Our second treatment scenario considered a female population with 90% responders and 10% non-responders to pesticide or sterility-inducer treatment, mating with males whose proportions of non-responders varied with the male mating systems described above (Fig. 6). While the average fitness of these females was reduced ten-fold compared to scenario 1, the variance in female fitness increased from 5- to 15-fold depending on the magnitude of *JK* (Tables 1b and 2a). Again, genetic monandry (GM = genetic monogamy, see above) returned male fitness parameters, *V*_*Omales(JK)*_ and *I*_*males(JK)*_, identical to those of females, the ratio *I*_*males(JK)*_/*I*_*females(JK)*_ again equaled 1 (Fig. 7a), and the values of *I*_*females(JK)*_, *I*_*males(JK)*_ and *I*_*average(JK)*_ were all equivalent (Fig. 8a; Table 2a, Appendix E).

Random mating (RM), moderate sexual selection, and extreme sexual selection each led to increasing magnitudes for the male fitness parameters as described in the previous scenario, particularly in the value of *I*_*males(JK)*_ (Table 2a, Appendix E). The maximum value of *I*_*males(JK)*_ (=197.1 for *I*_*males(5,5)*_, Table 2a) in this scenario was nearly twice that in scenario 1 (=99.2 for *I*_*males(5,5)*_, Table 2). However, increases in the ratio, *I*_*males(JK)*_/*I*_*females(JK)*_ were much less dramatic than in the first scenario, reaching only 2.01 at *J=K* = 15 (Fig. 7b; Table 2a, Appendix E). With female non-responders at 10%, the opportunity for selection on females, *I*_*females(JK)*_, exceeded 90.0 for all female fitness distributions. Thus, while smaller than *I*_*males(JK)*_, the average opportunity for selection between the sexes, *I*_*average(JK)*_, was 2–3 fold larger than in scenario 1 (90.0–147.6; Fig. 8b; Table 2a, Appendix E).

Our third treatment scenario considered a female population with 99% responders and 1% non-responders to pesticide or sterility inducer treatment mating with males whose proportions of non-responders varied with male mating system (Fig. 6). Again, the average fitness of these females was reduced by 10-fold compared to the previous scenario, with the variance in fitness increasing as the magnitude of *JK* increased (Table 1c, Appendix D; 2b, Appendix E). The values of *I*_*males(JK)*_ in this scenario were over 50-fold larger than in scenario 2 (Table 2a, Appendix E). However, in this scenario, increases in the ratio, *I*_*males(JK)*_/*I*_*females(JK)*_ were further reduced, reaching only 1.01 at J*=K* = 15 (Fig. 7c; Table 2b, Appendix E). Thus with 1% non-responders in each sex, despite intense within-sex selection, a sex difference in the opportunity for selection did not exist. The average opportunity for selection between the sexes, *I*_*average(JK)*_, however, was extremely large ranging from 9,904 to 9,953.5 (Fig. 8c; Table 2b, Appendix E).

In summary, the first three treatment scenarios, simulating increasingly larger proportions of the female and male pest populations being killed or sterilized by the treatment, showed that as the opportunity for selection on females and males increased, the magnitude of the average opportunity for selection (*I*_*average(JK)*_) favoring non-responsiveness also increased. However, the value of *I*_*average(JK)*_ was always intermediate between that of *I*_*males(JK)*_ and *I*_*females(JK)*_. Increasing opportunities for selection within each sex were possible because the variance in fitness within each sex increased, thus the magnitude of the sex difference in the opportunity for selection (*I*_*males(JK)*_/*I*_*females(JK)*_) by necessity decreased, and was negligible when selection on males and females was at maximum (1% non-responders for each sex).

The fourth scenario, like the third scenario above, also considered a female population with 99% responders and 1% non-responders to treatment, with males whose proportions of non-responders varied with male mating system (Fig. 6). However, these females were embedded within a population of responder females whose fecundity was reduced rather than eliminated by treatment with a contraceptive. As shown in section 2.7 above, embedding 1% non-responder females within populations of 99% responders with reduced fecundity, dramatically reduced the opportunity for selection on females favoring non-responsiveness to treatment (Fig. 5d; Table 2). We observed a similar result in this simulation (Table 3). However, layering mating systems with increasing variance in male mating success (GM, RM, SSM, SSE) onto this female fecundity distribution returned nearly identical results to those found for scenario 1 in the values of *I*_*females(JK)*_, *I*_*males(JK)*_ and *I*_*average(JK)*_ (Figs. 7a, d and 8a, d; Tables 2 and 3).

**Table 3:** The effect of mating system on the opportunity for sexual selection in a rat population with 1% female non-responders to treatment, embedded within a population of 99% responder females whose fertility is reduced but not eliminated by contraceptive.

Female fitness distribution	Female parameters			Male parameters	Male mating system			
(*J* = *V*_*J*_ = *K*=*V*_*K*_)	*O*_*females(JK)*_	*V*_*Ofemales(JK)*_	*I*_*females*_		GM	RM	SSM	SSE
5	27.15	146.40	0.199	*V*_*Omales*_	146.40	854.64	1,856.50	73,121.50
				*I*_*males*_	0.199	1.159	2.519	99.199
				*I*_*males*_/*I*_*females*_	1.00	5.83	12.68	499.46
				*I*_*average*_	0.20	0.68	1.36	49.70
10	98.30	965.30	0.100	*V*_*Omales*_	98.30	10,241.67	23,383.20	957,591.40
				*I*_*males*_	0.100	1.060	2.420	99.100
				*I*_*males*_/*I*_*females*_	1.00	10.61	24.22	922.01
				*I*_*average*_	0.10	0.58	1.26	49.60
15	223.80	3,108.30	0.062	*V*_*Omales*_	3,108.30	51,191.28	119,308.80	4,961,665.90
				*I*_*males*_	0.062	1.022	2.382	99.662
				*I*_*males*_/*I*_*females*_	1.00	16.47	38.38	1,596.26
				*I*_*average*_	0.06	0.54	1.22	49.86

GM = genetic monogamy; RM = random mating; SSM = moderate sexual selection; SSE = extreme sexual selection (details provided in text).

These latter results suggested that although embedding female non-responders within a population of female responders with reduced fecundity, reduced the opportunity for selection favoring non-responsiveness to treatment through females, compared to scenario 1, it had little effect on how the opportunity for selection favoring non-responsiveness to treatment operated through males. Nevertheless, the average opportunity for selection between the sexes favoring non-responsiveness to a contraceptive in scenarios 1 and 4, *I*_*average(JK)*_ (≈50; Tables 2 and 3; Fig. 8), was approximately half the value of *I*_*males(JK)*_ in these scenarios (≈99; Tables 2 and 3) and was considerably less than the value of *I*_*females(JK)*_ in the simulations in which responsive females were presumed to die or become sterile (≈90–9,900; Tables 1a–c, Appendix D). Thus, the opportunity for selection favoring non-responsiveness to a contraceptive with sex-specific effects (e.g., Mayer et al., 2002, 2004; Dyer et al., 2013; Dyer and Mayer, 2014; Pyzyna et al., 2014), was smallest, either when only males were affected by the treatment, or when males were affected and non-responsive females were embedded within populations of female responders with reduced fecundity.

A further consideration is that in scenarios 1–4, the greatest effects of males on the average opportunity for selection favoring non-responsiveness occurred under moderate to extreme sexual selection (SSM, SSE), mating systems in which the covariance between mating success and non-responsiveness to treatment was assumed to equal 1. That is, only males that were non-responsive to the treatment were successful in siring offspring. This is certain to be the default condition for pesticides, and tight covariance between mating success and non-responsiveness to treatment is likely to rapidly evolve for treatments with sterility inducers (see below). However, for contraceptives, if this covariance is assumed to be weaker, or if no association is assumed to exist between mating success and non-responsiveness, then random mating (RM) might accurately describe how selection favors non-responsiveness. In all cases for this scenario, the average opportunity for selection between the sexes, *I*_*average(JK)*_, under RM was less than 1 (Table 3, Fig. 8d), similar to a condition in which selection occurs by chance alone.

### 3.6 Assortative mating and the evolution of resistance to pesticides, sterility-inducers and contraceptives

Within each of the four scenarios used to illustrate variation in the intensity of pesticide or sterility inducer treatment, female preferences for particular males increased the sex difference in the opportunity for selection, *I*_*mates(preferred)*_. Responses in *I*_*mates(preferred)*_ ranged from 1.02–6.83 (Fig. 9a, d) to 9,900.4–56,574.0 (Fig. 9c), indicating that changes in the sex differences in the opportunity for selection as a result of female mate choice, could become very large, particularly when the fraction of females responding to treatment was high (Fig. 9b, c). These results indicate that female preferences for particular males could further modify the opportunity for selection favoring non-responsiveness to pesticides, sterility inducers and contraceptives, beyond the opportunity for selection arising from the application of these treatments (Figs. 5, 7 and 8). In particular, if positive assortative mating occurred, the opportunity for selection favoring non-responsiveness could be enhanced from 1- to 6-fold. Note that this increase only considers the initial effect of positive assortative mating and does not consider the accelerating influence of genetic correlations among heritable traits that would be established when such non-random mating occurs (Lande, 1981; Kirkpatrick, 1982; Shuster and Wade, 2003). If negative assortative mating occurred, the opportunity for selection favoring non-responsiveness would be diminished by an equivalent amount.

## 4 Discussion

Our simulations used published information on life history traits in female rats (Knipling and McGuire, 1972; Clark and Price, 1981; Sridhara and Krishnamurthy, 1992; Keiner, 2005) to provide a method for visualizing female fecundity in natural populations. This approach allowed us to generate an explicit description of how the variance in relative fitness among females, i.e., the opportunity for selection (Crow, 1958, 1962), can be estimated using this information. Our approach provided an estimate of the magnitude of opportunity for selection imposed by pesticides and sterility-inducers designed to eliminate their target pest populations. Compared to selection occurring by chance alone, our simulations showed that the opportunity for selection favoring resistance, following the application of pesticides and sterility-inducers, can increase by five orders of magnitude (165 thousand-fold) given the life history information we used and the assumptions we made about pesticide and sterility inducer effects. Because such treatments seldom if ever completely extirpate a pest population, our estimates provide an explicit estimate of the intensity of total selection likely to favor the evolution of traits conferring resistance to these treatments. We note that our estimate is likely to be conservative because we only considered scenarios in which the maximum effectiveness of our hypothetical pesticide was 99%, and we did not consider genetic correlations that could influence selection intensity. Higher pesticide effectiveness is cited for most studies (Knipling, 1959; Knipling and McGuire, 1972; Talpaz et al., 2010). Thus, our framework could allow estimates of the intensity of selection favoring resistance to pesticides and sterility inducers in other pest species.

Our simulations did not distinguish between responder individuals who were sterilized or were killed by the treatment. We did not address this issue because we were specifically interested in examining the effect of selection favoring resistance, which would operate through reproducing individuals, regardless of how responders were eliminated. More importantly, our results show that attempts to control pest populations by releasing sterile individuals (e.g., Harris, 2017) are likely to be ineffective, in part because selection for resistance can still operate through the non-sterile sex (as indicated by studies showing the evolution of female discrimination of sterile males (Hibino and Iwahashi, 1991; Pérez-Staples et al., 2013), and in part because selection favoring female discrimination can be accelerated through female preferences for non-sterile males. Our results provide an explanation for why this process may operate so quickly, and they cast further doubt on the effectiveness of past and recent efforts to use this approach (Knipling, 1959; Krasfur, 1998; Klassen and Curtis, 2015; Ant et al., 2012; Perez-Staples et al., 2013; USDA report 2016; Balestrino et al., 2017). Additional sources of selection from predation or interference competition could act synergistically with treatment that merely sterilized responders to reduce population size, but we did not consider this possibility in detail.

Our simulations showed too that although the mean and variance in offspring numbers increased as litter size and litter number increased, the opportunity for selection on females decreased (Fig. 4b). This result seems paradoxical given that increasing female fecundity increases the variance in female fitness disproportionately to the average in female fitness. However, because the opportunity for selection equals the variance in fitness divided by the squared average in fitness, and because the squared average in offspring numbers increases faster than the variance, increases in the mean and variance in offspring numbers reduced the opportunity for selection on females.

Although the effect of litter number on the opportunity for selection appeared to be slightly greater than litter size when litter number was small (*J* = 5; Fig. 4b), most of our results suggested that the relative influences of litter size and litter number on the opportunity for selection were similar. This latter result is consistent with ecological theory suggesting that the relative influences of litter size and litter number on fertility selection and thus on the evolution of life history strategies can be approximately equivalent (Cole, 1954). However, it is possible that the relative contributions of litter size and number would differ with more variable life histories. Again, this framework provides a means for further exploration.

As the fraction of non-responders to pesticides and sterility-inducers decreased within our simulated populations, the opportunity for selection favoring this trait increased. Specifically, for every order of magnitude decrease in the frequency of non-responders, a condition equivalent to treatment with more powerful or more effective pesticides and sterility inducers, our simulations generated a two to three order of magnitude increase in the opportunity for selection favoring resistance (Fig. 5; Table 1, 1a–d, Appendix D). Because our hypothetical population consisted of only 100 females, only two comparisons of the relationship between the proportion of non-responders in the pest population and the increase in the opportunity for selection were possible (10%, 1%). However, the result suggests that this relationship could be extrapolated to circumstances with larger untreated population sizes and smaller fractions of survivors after treatments (c.f., Knipling, 1959; Knipling and McGuire, 1972). Our results also show that the combined use of pesticides or sterility inducers, with contraceptives will be ineffective. While reductions in fertility alone can reduce selection favoring non-responders, this effect is eliminated if responders are then removed from the breeding population entirely by pesticides and sterility inducers.

How realistic are these estimates? In natural populations, most estimates of the opportunity for selection are less than 0.5 and only in situations with large skews in fitness variance do larger values appear. For example, values for the opportunity for selection due to differences in mating success among males have maxima less than 9.0 (Jones et al., 2001, Table 3, *I*_*males*_ = 0.25–2.03; Wade and Shuster, 2004, Table 1, *I*_*males*_ = 1.36–8.99; Tatarenkov et al., 2008, Table 4, *I*_*males*_ = 0.16–4.52). Our maximum values for the opportunity for selection on females, *I*_*females*_, due to the effects of pesticides or sterility inducers, were larger than these estimates by three orders of magnitude (9,904.0, Table 1, Fig. 5c). Our estimates of *I*_*males*_ for males mating with females under selection from pesticides or sterility inducers, with extreme skew in mating success (SSE) designed to simulate the effects of such treatment, were larger than the above field estimates of *I*_*males*_ by four orders of magnitude (10,003.0, Table 2b; Fig. 5c). The differences in our estimated values and those of field estimates of sexual selection are in part due to the fact that individual males are seldom able to mate with all of the females in a natural population, which our simulations allowed. However, because our simulations show similarity between the action of sexual selection and selection resulting from the application of pesticides and sterility inducers, they underscore the conclusion that the conditions favoring the evolution of pesticide resistance are powerful indeed.

Our results also reveal a novel approach for the control of pest populations, and possibly for the application of antibiotics to control pathogens (Hughes and Andersson, 2012). Although a population of females containing 1% non-responders generated the largest opportunities for selection favoring pesticide resistance, when non-responders were embedded within female populations who responded to treatment, not by dying or becoming sterile, but instead with reduced fecundity, as might be induced by a contraceptive, the opportunity for selection favoring resistance decreased dramatically, returning variation in the mean and variance in fitness and in the opportunity for selection on females, to values approximately equivalent to simulated populations in which no treatment was applied (Fig. 5d; Table 1d, Appendix D). This result is striking because it verifies an alternative paradigm for controlling pest populations, and could also provide a means for controlling invasive species as well. Although the existence of non-responders within a population of individuals who respond to treatment with reduced fecundity could allow the evolution of non-responsiveness, the rate at which such resistance evolves is reduced to that which is possible by chance alone.

The apparent explanation for the return of extreme values for the opportunity for selection, to values similar to those expected by chance, is consistent with classical life history and with population genetic theory (Cole, 1954; Kimura, 1962). When responders are eliminated from the population by pesticides, the remaining population consists entirely of non-responders. Lacking conspecific competitors and surrounded by abundant resources, such populations are expected to rapidly rebound (Fig. 1), and evidently, they usually do. Sterility inducers allow responders to persist within the population and thus may contribute to density-dependent processes that suppress reproduction by non-responders (Wolff, 2003). However, because sterilized individuals do not reproduce, selection favoring responders will still proceed with the same intensity that would exist if lethal treatment had been applied. In contrast, when responders, with their fecundities reduced by contraceptives, also remain within the population in their pre-treatment proportions, non-responders, despite their apparent fitness advantage, represent only a tiny fraction of the total population. In a world where bad things can happen to good genes and vice versa, when non-responders are rare, their probability of increasing in frequency is surprisingly small, despite their seemingly large fitness advantage (Wade and Shuster, 2010).

Immediate control of pest populations whose fecundity is reduced by contraceptives, will be less obvious than when the pest population is initially laid waste by more lethal measures of control. However, reductions in population size caused by reduced fecundity may be more effective in controlling pests than is now realized. Responders who remain within the population will not be replaced only by non-responders, allowing existing enemies and predators to reduce these populations as described above. We did not specifically model this process, but fundamental ecological principles predict that populations whose birth rates are reduced and whose mortality rates remain unchanged will rapidly decrease in size (Cole, 1954; Knipling, 1959). While such a process may be slower than the cataclysmic population declines produced by lethal pesticides, in the long run some morbidity and moribundity among hosts or crops may be preferable to the inevitable outcome of pest reductions using pesticides; i.e., cycles of resurgence by increasingly virulent pests, met with increasingly toxic chemicals, that favor increasing pest resistance to human control.

Our simulations of the sex-specific responses of rats to pesticides, sterility-inducers and contraceptives explored not only the effects of selection on resistance within each sex, but also confirmed two aspects of sexual selection theory. First, our simulations showed that under genetic monandry (GM; which here was equivalent to genetic monogamy; see section 3.5), female and male fitness and parameters associated with selection were identical (Shuster and Wade, 2003). This is true because under genetic monogamy, male and female fitnesses are explicitly linked. Second, as the variance in fitness among females increased due to differences in offspring numbers among females, this increase eroded the variance in male fitness due to differences in mating success among males (Wade, 1979; Shuster et al., 2013). Thus, when the opportunity for selection favoring non-responsiveness to pesticides and sterility-inducers was most extreme in both sexes, the sex difference in the opportunity for selection (i.e., sexual selection) was negligible. These two results conform to sexual selection theory and substantiate our use of this approach for understanding sex differences in responsiveness to pesticide treatment.

Within this framework, our simulations also showed that when males and females respond differentially to pesticides, sterility-inducers and contraceptives, the average opportunity for selection favoring non-responsiveness was less than if both sexes are equally responsive to the treatment. In particular, while embedding non-responsive females within populations of females with reduced fecundity had little effect on how selection favoring non-responsiveness acted through males, the average opportunity for selection favoring non-responsiveness was still reduced because of the reduction in the opportunity for selection through females. This result indicates that a sex difference in responsiveness to contraceptives may require different strategies for decreasing selection intensity with in each sex. However, this result also suggests that a sex difference in responsiveness to contraceptives can be exploited to weaken overall selection favoring resistance to these treatments.

A sex difference in the responsiveness of populations to treatment with pesticides, sterility-inducers and contraceptives suggests other approaches for mitigating evolved resistance as well. Our simulations assumed that only males who were non-responsive to treatment were successful in siring offspring, and vice versa. Stated differently, the covariance between non-responsiveness to pesticide treatment and male mating success was assumed to be equal to, or nearly equal to 1. This assumption is the basis for the evolution of exaggerated traits via sexual selection (Lande, 1981; Kirkpatrick, 1982; Shuster and Wade, 2003). This assumption also explains why the opportunity for selection favoring non-responsiveness to treatment could continue to increase with increasing proportions of responder males. However, if this covariance is weakened or if it is eliminated entirely, the opportunity for selection favoring non-responsiveness to the treatment through males disappears. One possible means to accomplish this is to develop contraceptives that reduce rather than eliminate male fertility similar to how the contraceptives now decrease the fertility of females.

We found that assortative matings among females and males who are responsive and non-responsive to treatment using pesticides, sterility-inducers and contraceptives can also influence the opportunity for selection favoring non-responsiveness. How strongly selection favoring non-responsiveness acts on males and females within a population will determine the influence assortative mating has on the overall opportunity for selection. If positive assortative mating occurs, the opportunity for selection favoring non-responsiveness could be enhanced by as much 6-fold beyond that caused by the selection produced by pesticides or sterility inducers. This effect could further increase if the few non-responders remaining in a treated population tended to prefer one another as mates, and could continue to accelerate if preferences lead to genetic correlations between male and female traits. This result argues against the continued use of sterile male procedures for controlling pests (e.g., in Drury et al., 2017; Harris, 2017). In contrast, if negative assortative mating occurs, not only is the opportunity for selection due to mate preferences expected to oppose the opportunity for selection favoring non-responsiveness through fertility selection, but the relative numbers of responders preferring to mate only with non-responders or vice versa will determine how strong this opposition becomes. The formation of negative genetic correlations between male and female responsiveness as a result of mate choices could further slow evolved resistance.

Overall, our results indicate that pest control measures that reduce fertility, rather than those that cause sterilization or death in pests, are likely to be most effective in slowing the evolution of treatment resistance. Furthermore, fertility-reducing treatments that work on both sexes will be most effective in reducing pest population size, and in mitigating selection favoring treatment resistance. When sex-dependent effects of pest treatments exist, the opportunity for selection favoring non-responders will be lowest, again if both sexes are affected and if neither sex becomes sterile or dies after treatment. If this is unavoidable, sterility is less likely to favor non-responders than death because sterile individuals could still remain in the population and successfully mate, thus interfering with reproduction by fertile non-responders. However, this latter effect is likely to be transitory because mate choices that actually produce offspring will be favored, and eventually a covariance between non-responders and fertility is expected to arise and be further enhanced by particular mate preferences, as we have shown (Fig. 9). While negative assortative mating could slow this process, positive assortative mating, rather than negative assortative mating associated with responsiveness is more likely to arise (Shuster and Wade, 2003; Drury et al., 2017).

Our proposed approach to pest control is a considerable departure from existing methodologies, which attempt to remove unwanted species from the environment entirely. The possibility exists that an emphasis on fertility reduction rather than population extirpation is impractical because some level of disease and damage will exist, even in treated populations. However, we suggest that persistent but lower levels of damage caused by pests and pathogens is preferable to continued acceleration of evolved resistance. We cannot know which solution is less onerous until we try a different path.

## Declarations

### Author contribution statement

Stephen M. Shuster, Brandy Pyzyna, Loretta P. Mayer, Cheryl A. Dyer: Conceived and designed the experiments; Performed the experiments; Analyzed and interpreted the data; Contributed reagents, materials, analysis tools or data; Wrote the paper.

### Funding statement

This research did not receive any specific grant from funding agencies in the public, commercial, or not-for-profit sectors.

### Competing interest statement

The authors declare the following conflict of interests: Brandy Pyzyna, Loretta Mayer, and Cheryl Dyer are employees of Senestech, Inc., a company that manufactures and markets a pest control product whose mechanism of action is through a reduction in male and female fertility; Stephen M. Shuster is a paid consultant to Senestech, Inc.

### Additional information

No additional information is available for this paper.
