# Supplementary material for: The opportunity for sexual selection and the evolution of non-responsiveness to pesticides, sterility inducers and contraceptives
Source: Heliyon. 2018 Nov 29;4(11):e00943. doi: 10.1016/j.heliyon.2018.e00943 (PMC6275691; doi:10.1016/j.heliyon.2018.e00943)
Supplement: Appendix A [file mmc1.docx]

Appendix A

Simulations of Rat Reproduction – the Effects of Litter Number

We generated a normal distribution for the number of litters produced by 100 hypothetical female rats, reproducing with an average litter number, *J*, and a variance in litter number, *V_J_*, equal to 15 litters. To approximate reproduction by individual females in this initially continuous distribution, we identified 30 litter number classes by rounding the numbers of females contained within each *j*-th litter number class to integer values, while keeping the mean, *J*, and variance, *V_J_*, in litter number at 15 litters per female, and the total sample size at 100 females.

We confirmed the accuracy of this approach by empirically estimating the average litter number, *J*, as the number of females reproducing within each *j*-th litter class, *f_j_*, multiplied by the number of litters in each *j*-th litter number class, *l_j_*, summed over all *j* litter classes, and divided by the number of females in each *j*-th litter class, summed over all *j* litter classes, or,

*J* = (Σ *f_j_* *l_j_*) / (Σ *f_j_*). (A.1)

Similarly, we confirmed that the variance in litter number, *V_J_*, for this distribution equaled the average of the squared number of litters produced by females, minus the square of the average number of litters produced by females. Stated differently, *V_J_* equaled the number of females in each *j*-th litter number class, *f_j_*, multiplied by the squared number of litters in each *j*-th litter number class, *l_j_*^2^, summed over all *j* litter number classes, and divided by the number of females in each *j*-th litter number class, summed over all *j* litter number classes; from this quantity we subtracted the squared average number of litters per female, *J*, as estimated in Eq. 1, or,

*V_j_* = [(Σ *f_j_* *l_j_*^2^ ) / (Σ *f_j_*)] – [Σ *f_j_* *l_j_* / Σ *f_j_*]^2^ (A.2)

The range of the distribution of litter numbers included females producing a minimum of *j*=7 litters, to females producing a maximum of *j*=24 litters. We performed similar procedures to generate normal distributions of 100 female litter numbers with a mean (*J*) and variance (*V_J_*) equal to 10 and 5 litters per female respectively. The ranges in litter number for these distributions were *j* = 3-18 litters per female and *j* = 0-11 litters per female, respectively (Fig. 3a-c).
