# Supplementary material for: The opportunity for sexual selection and the evolution of non-responsiveness to pesticides, sterility inducers and contraceptives
Source: Heliyon. 2018 Nov 29;4(11):e00943. doi: 10.1016/j.heliyon.2018.e00943 (PMC6275691; doi:10.1016/j.heliyon.2018.e00943)
Supplement: Appendix B [file mmc2.docx]

Appendix B

Simulations of Rat Reproduction – the Effects of Litter Size

We generated a normal distribution of 100 hypothetical female rats reproducing with an average litter size, *K*, and a variance in litter size, *V_K_*, equal to 15 offspring per litter. To approximate reproduction by individual females in this initially continuous distribution, we identified discrete numbers of females within 30 litter size classes by rounding the numbers of females contained within each *k*-th litter size class to integer values, keeping the mean, *K*, and variance, *V_K_*, in litter number at 15 offspring per litter per female, and the total sample size at 100 females.

We confirmed the accuracy of this approach by empirically estimating the average litter size, *K*, as number of females producing each *k*-th litter size class, *f_k_*, multiplied by the number of offspring in each *k*-th litter size class, *o_k_*, summed over all *k* litter classes, and divided by the number of females in each *k*-th litter size class, summed over all *k* litter size classes, or

*K* = (Σ *f_k_* *o_k_*) / (Σ *f_k_*). (B.1)

Similarly, we confirmed that the variance in litter size, *V_K_*, for this distribution equaled the average of the squared litter size produced by females, minus the square of the average litter size produced by females; that is, the number of females in each *k*-th litter size class, *f_k_*, multiplied by the squared number of offspring in each *k*-th litter size class, *o_k_*^2^, summed over all *k* litter size classes, and divided by the number of females in each *k*-th litter size class, summed over all *k* litter size classes; from this quantity we subtracted the squared average litter size per female, *K*, as estimated in Eq. 3, or,

*V_K_* = [(Σ *f_k_* *o_k_*^2^ ) / (Σ *f_k_*)] – [Σ *f_k_* *o_k_* / Σ *f_k_*]^2^ (B.2)

The range of this distribution included females producing a minimum of *k*=7 offspring per litter to females producing a maximum of *k*=24 offspring per litter. We performed similar procedures to generate normal distributions of 100 female litter numbers with a mean (*K*) and variance (*V_K_*) of 10 and 5 offspring per litter per female respectively. The ranges in litter size for these distributions were *k* = 3-18 offspring per litter per female and *k* = 0-11 offspring per litter per female, respectively (Fig. 3a-c).
