# Supplementary material for: The opportunity for sexual selection and the evolution of non-responsiveness to pesticides, sterility inducers and contraceptives
Source: Heliyon. 2018 Nov 29;4(11):e00943. doi: 10.1016/j.heliyon.2018.e00943 (PMC6275691; doi:10.1016/j.heliyon.2018.e00943)
Supplement: Appendix C [file mmc3.docx]

Appendix C

Quantifying the Mean and Variance in Female Offspring Numbers

We estimated the average number of offspring for each combined distribution as *O_females(JK)_*, where *J* represented the average number of litters per female (=15, 10, 5) and *K* represented the average litter size (number of offspring per litter; =15, 10, 5), as the weighted sum of the product of the number of litters produced by females in each litter number class, and the average litter size expressed as the number of offspring per litter for each litter size distribution, or,

*O_females(JK)_* = Σ *p_j_* (*j K*), (C.1)

where *p_j_* equaled the proportion of females in the sample of 100 females belonging to each *j*-th litter number class, such that *p_j_* = *f_j_* / (Σ *f_j_*), *j* equaled the number of litters produced by females in the *j*-th litter number class, and *K* equaled the average number of offspring per litter in the *K*-th average litter size distribution.

We next partitioned the total variance in offspring numbers produced by females, *V_Ofemales(JK)_*, into within-female, *V_Owithin(JK)_*, and among-female, *V_Oamong(JK)_*, components such that,

*V_Ofemales(JK)_* = *V_Owithin(JK)_* + *V_Oamong(JK)_*, (C.2)

where *JK* represented the combined distribution in which the average number of litters per female equaled *J* (=15, 10, 5) and the average litter size equaled *K* (=15, 10, 5).

As shown in similar form elsewhere (Wade 1979; Shuster and Wade 2003; Wade and Shuster 2004), when females differ in litter number, the within-females component of the total variance in female fitness, *V_Owithin(JK)_*, equals the weighted average of the variance in female offspring numbers, summed across all *j* litter number classes, within the *JK*-th litter size distribution, or,

*V_Owithin(JK)_* = Σ *p_j_* (*j V_(JK)_*), (C.3)

where *p_j_* equals the fraction of females in the sample of 100 females belonging to each *j*-th litter number class, *j* equals the number of litters that females in each class produce, and *V_(JK)_* equals the variance in litter numbers within the sample of 100 females, with the *J*-th average number of litters per female and the *K*-th average litter size per female.

The among-females component of the total variance in female fitness due to differences in litter number, equals the variance of the average female offspring numbers, estimated across all *j* litter number classes, or,

*V_Oamong(JK)_* = Σ *p_j_* (*jK* - *JK*)^2^, (C.4)

where *p_j_* equals the fraction of females in the sample of 100 females belonging to each *j*-th litter number class, *j* equals the number of litters that females in each litter class produced, *K* equals the average litter size in the *K*-th average litter size distribution and *J* equals the average number of litters per female in the *J*-th average litter number distribution. Thus, the total variance in offspring numbers produced by females equaled,

*V_Ofemales(JK)_* = Σ *p_j_* (*j V_(JK)_*) + Σ *p_j_* (*jK* - *JK*)^2^ (C.5)

To simulate the effect of variable litter number on rat reproduction, for each of the first set of distributions in which the average litter number, *J* = 15, 10, and 5, we allowed the average litter size, *K*, and variance in litter size, *V_K_*, to equal 15 offspring per litter. To simulate the effect of variable litter size on rat reproduction, for each of the second set of distributions, in which the average litter size, *K* = 15, 10 and 5 offspring per litter per female, we allowed the average litter number, *J*, and variance in litter number, *V_J_*, to equal 15 litters per female. To examine the combined effect of litter number and litter size on rat reproduction on this range of litter numbers and litter sizes, we generated a 3x3 matrix containing these and all remaining hypothetical distributions of 100 female rats in which the average and variance in litter number, *J*, and *V_J_*, and the average and variance in litter size, *K* and *V_K_*, equaled 15, 10 and 5, respectively (Table 1). We then plotted the resulting distributions to show the total range of variation in offspring number within our simulations (Fig. 4a).
