# Supplementary material for: The opportunity for sexual selection and the evolution of non-responsiveness to pesticides, sterility inducers and contraceptives
Source: Heliyon. 2018 Nov 29;4(11):e00943. doi: 10.1016/j.heliyon.2018.e00943 (PMC6275691; doi:10.1016/j.heliyon.2018.e00943)
Supplement: Appendix D [file mmc4.docx]

Appendix D.

The Effect of Non-responders on Rat Reproduction

We examined the effect that most pest control measures have on rat reproduction; specifically, procedures designed to kill or sterilize individuals and thus severely reduce the overall size of the reproducing population of pests. To simulate this process, we began by identifying three female fitness distributions by modifying the fractions of reproducing individuals generated in the last experiment as follows. To examine the effect of 90% responders and 10% non-responders (i.e., 90% of the population removed or sterilized by the pesticide or sterility inducer, and 10% of the population unaffected by the treatment), we allowed 10/100 females with the average and variance in litter number, *J* and *V_J_*, and average and variance in litter size, *K* and *V_K_*, equaling 15, 10, and 5, respectively, to successfully reproduce; all other females in the population were assumed to produce no offspring. To examine the effect of 95% responders and 5% non-responders (i.e., 95% of the population removed or sterilized by the pesticide or sterility inducer, and 5% of the population unaffected by the treatment), we allowed 5/100 females with the average and variance in litter number, *J* and *V_J_*, and average and variance in litter size, *K* and *V_K_*, equaling 15, 10, and 5, respectively, to successfully reproduce; all other females in the population were assumed to produce no offspring. To examine the effect of 99% responders and 1% non-responders (i.e., 99% of the population removed or sterilized by the pesticide or sterility inducer, and 1% of the population unaffected by the treatment), we allowed 1/100 females with the average and variance in litter number, *J* and *V_J_*, and average and variance in liter size, *K* and *V_K_*, equaling 15, 10, and 5, respectively, to successfully reproduce; all other females in the population were assumed to produce no offspring. As mentioned in the text, variation in the proportions of responders and non-responders, combined with reduction in female fecundity provides a means for examining the effects of the simultaneous application of a pesticide or sterility inducer, with a contraceptive. However, to facilitate tabulation and graphing, the results are presented as *JK* = 5, 10, 15.

| Table 1a. The effect of 10% non-responders on the mean (*O_females(JK)_*) and variance (*V_Ofemales(JK)_*) in offspring numbers and the opportunity for selection on females (*I_females_*); the first row of each cell contains the mean and variance (in parentheses); the second row contain *I*. | | | | | |
| --- | --- | --- | --- | --- | --- |
|  |  |  |  |  |  |
|  |  | Litter number (*J*) |  |  |  |
|  |  |  |  |  |  |
| Litter size (*K*) | | 5 | 10 | 15 |  |
|  |  |  |  |  |  |
|  | 5 | 2.40 (565.0) | 5.0 (2255.0) | 7.5 (5070.0) |  |
|  |  | 98.09 | 90.20 | 90.13 |  |
|  |  |  |  |  |  |
|  | 10 | 5.0 (2255.0) | 10.0 (9010.0) | 15.0 (20265.0) |  |
|  |  | 90.20 | 90.10 | 90.07 |  |
|  |  |  |  |  |  |
|  | 15 | 7.5 (5070.0) | 15.0 (20265.0) | 22.5 (45585.0) |  |
|  |  | 90.13 | 90.07 | 90.04 |  |

| Table 1b. The effect of 5% non-responders on the mean (*O_females(JK)_*) and variance (*V_Ofemales(JK)_*) in offspring numbers (first row) and the opportunity for selection on females (*I_females_*); the first row of each cell contains the mean and variance (in parentheses); the second row contain *I*. | | | | | | | | |
| --- | --- | --- | --- | --- | --- | --- | --- | --- |
|  |  | |  | |  | |  |  |
|  | Litter number (*J)* | |  | |  | |  |  |
|  |  | |  | |  | |  |  |
| Litter size (*K*) | | 5 | | 10 | | 15 | |  |
|  |  | |  | |  | |  |  |
| 5 | 1.3 (595.0) | | 2.5 (2372.5) | | 3.8 (5347.5) | |  |  |
|  | 380.8 | | 380.4 | | 380.3 | |  |  |
|  |  | |  | |  | |  |  |
| 10 | 2.5 (2372.5) | | 5.0 (9505.0) | | 7.5 (21382.5) | |  |  |
|  | 380.4 | | 380.2 | | 380.13 | |  |  |
|  |  | |  | |  | |  |  |
| 15 | 3.8 (5347.5) | | 7.5 (21382.5) | | 11.3 (48105.0) | |  |  |
|  | 380.3 | | 380.13 | | 380.1 | |  |  |

| Table 1c. The effect of 1% non-responders on the mean (*O_females(JK)_*) and variance (*V_Ofemales(JK)_*) in offspring numbers (first row) and the opportunity for selection on females (*I_females_*); the first row of each cell contains the mean and variance (in parentheses); the second row contain *I*. | | | | | | | | |
| --- | --- | --- | --- | --- | --- | --- | --- | --- |
|  |  | |  | |  | |  |  |
|  | Litter number (*J*) | |  | |  | |  |  |
|  |  | |  | |  | |  |  |
| Litter size (*K*) | | 5 | | 10 | | 15 | |  |
|  |  | |  | |  | |  |  |
| 5 | 0.25 (619.0) | | 0.50 (2475.5) | | 0.75 (5569.5) | |  |  |
|  | 9904.0 | | 9902.0 | | 9901.3 | |  |  |
|  |  | |  | |  | |  |  |
| 10 | 0.50 (2475.5) | | 1.00 (9901.0) | | 1.50 (22276.5) | |  |  |
|  | 9902.0 | | 9901.0 | | 9900.67 | |  |  |
|  |  | |  | |  | |  |  |
| 15 | 0.75 (5569.5) | | 1.50 (22276.5) | | 2.25 (50121.0) | |  |  |
|  | 9901.3 | | 9900.67 | | 9900.44 | |  |  |

| Table 1d. The effect of 1% non-responders embedded within populations of responders, with fecundities reduced by contraceptive, on the mean (*O_females(JK)_*) and variance (*V_Ofemales(JK)_*) in offspring numbers (first row) and the opportunity for selection on females (*I_females_*); the first row of each cell contains the mean and variance (in parentheses); the second row contains *I*. | | | | | | | | |
| --- | --- | --- | --- | --- | --- | --- | --- | --- |
|  |  | |  | |  | |  |  |
|  | Litter number (*J*) | |  | |  | |  |  |
|  |  | |  | |  | |  |  |
| Litter size (*K*) | | 5 | | 10 | | 15 | |  |
|  |  | |  | |  | |  |  |
| 5 | 27.15 (146.4) | | 49.10 (265.9) | | 74.6 (395.1) | |  |  |
|  | 0.199 | | 0.110 | | 0.071 | |  |  |
|  |  | |  | |  | |  |  |
| 10 | 52.05 (529.1) | | 98.30 (965.3) | | 149.20 (1431.2) | |  |  |
|  | 0.195 | | 0.100 | | 0.064 | |  |  |
| 15 | 76.95 (1150.2)  0.194 | | 147.45 (2098.2)  0.097 | | 223.80 (3108.3)  0.062 | |  |  |
