# Supplementary material for: The opportunity for sexual selection and the evolution of non-responsiveness to pesticides, sterility inducers and contraceptives
Source: Heliyon. 2018 Nov 29;4(11):e00943. doi: 10.1016/j.heliyon.2018.e00943 (PMC6275691; doi:10.1016/j.heliyon.2018.e00943)
Supplement: Appendix E [file mmc5.docx]

Appendix E

Using Mating System to Examine the Magnitude of the Sex-Specific Effect of Selection on Non-responders

For each of the four female scenarios described in Appendix D, we generated four mate-number distributions for males that varied in the degree to which each produced a sex difference in opportunity for selection (Fig. 6). Within each distribution we varied the proportion of the male population within which responder males were assumed to die or become sterile as a result of treatment with a pesticide or sterility inducer, as well as the mate numbers obtained by non-responder males who were assumed to be unaffected by the treatment and thus were able to mate with females. The distributions of non-responder males were defined by the following mating systems; (1) genetic monandry (GM; 100% non-responders) in which all males were presumed be non-responsive to treatment and to mate with only one female and to refuse to mate with non-virgin females; note that because each male in this treatment mates with only one unmated female, females also must mate with only one male; we assumed that all females would successfully mate; (2) random mating (RM; 64% non-responders, 36% responders) in which non-responder males were presumed to mate randomly with females; (3) moderate sexual selection (SSM; 46% non-responders, 54% responders) in which the mating success of non-responder males was moderately skewed, and (4) extreme sexual selection (SSE; 1% non-responders, 99% responders) in which the mating success of responder males was maximally skewed (Fig. 6).

Note that in mating system 2 (RM, random mating), 36% of the males did not mate due to chance alone, simultaneously illustrating the consequence of a treatment that affected males randomly within the population, as well as the effect that chance has on male mating success (Fig. 6). In this latter case there was no assumption that non-responsiveness correlated with male mating success (although such a covariance could be specified). However, in mating systems 4 and 5 (SSM, SSE, moderate and extreme sexual selection, respectively), non-responsiveness was presumed to covary explicitly with male mating success. Also note that because variation in female litter size and litter number were used to generate the distributions of female fertility, and because of the multiplicative relationship between male mate numbers and the mean and variance in female offspring numbers, these sources of variance in female fitness were included within the distributions of male fitness variance due to variation in male mate numbers (Wade 1979; Shuster and Wade 2003; Shuster et al. 2013).

| Table 2a. The effect of mating system on the opportunity for sexual selection in a rat population with 90% female responders to treatment; | | | | | | | | | | | |
| --- | --- | --- | --- | --- | --- | --- | --- | --- | --- | --- | --- |
| GM=genetic monogamy; RM=random mating; SSM=moderate sexual selection; SSE=extreme sexual selection (details provided in text). | | | | | | | | | | |  |
|  |  |  |  |  |  |  |  |  |  |  |  |
|  | Female fitness | Female |  |  |  | Male mating system | |  |  |  |  |
|  | distribution | parameters | |  | Male |  |  |  |  |  |  |
|  | (*J*=*V_J_*=*K*=*V_K_*) | *O_females(JK)_* | *V_Ofemales(JK)_* | *I_females_* | Parameters | GM | RM | SSM | SSE |  |  |
|  |  |  |  |  |  |  |  |  |  |  |  |
|  | 5 | 2.40 | 565.00 | 98.090 | *V_Omales_* | 565.00 | 570.53 | 578.40 | 1,135.20 |  |  |
|  |  |  |  |  | *I_males_* | 98.090 | 99.050 | 100.410 | 197.090 |  |  |
|  |  |  |  |  | *I_males_* / *I_females_* | 1.00 | 1.01 | 1.02 | 2.01 |  |  |
|  |  |  |  |  | *I_average_* | 98.09 | 98.57 | 99.25 | 147.59 |  |  |
|  |  |  |  |  |  |  |  |  |  |  |  |
|  | 10 | 10.10 | 9,010.00 | 90.100 | *V_Omales_* | 9,010.00 | 9,106.00 | 9,242.00 | 18,910.00 |  |  |
|  |  |  |  |  | *I_males_* | 90.100 | 91.060 | 92.420 | 189.100 |  |  |
|  |  |  |  |  | *I_males_* / *I_females_* | 1.00 | 1.01 | 1.03 | 2.10 |  |  |
|  |  |  |  |  | *I_average_* | 90.10 | 90.58 | 91.26 | 139.60 |  |  |
|  |  |  |  |  |  |  |  |  |  |  |  |
|  | 15 | 22.50 | 45,585.00 | 90.040 | *V_Omales_* | 45,585.00 | 3,108.30 | 46,759.50 | 95,703.80 |  |  |
|  |  |  |  |  | *I_males_* | 90.044 | 90.044 | 92.364 | 189.044 |  |  |
|  |  |  |  |  | *I_males_* / *I_females_* | 1.00 | 1.00 | 1.03 | 2.10 |  |  |
|  |  |  |  |  | *I_average_* | 90.04 | 90.04 | 91.20 | 139.54 |  |  |

| Table 2b. The effect of mating system on the opportunity for sexual selection in a rat population with 99% female responders to treatment; | | | | | | | | | | | |
| --- | --- | --- | --- | --- | --- | --- | --- | --- | --- | --- | --- |
| GM=genetic monogamy; RM=random mating; SSM=moderate sexual selection; SSE=extreme sexual selection (details provided in text). | | | | | | | | | | |  |
|  |  |  |  |  |  |  |  |  |  |  |  |
|  | Female fitness | Female |  |  |  | Male mating system | |  |  |  |  |
|  | distribution | parameters | |  | Male |  |  |  |  |  |  |
|  | (*J*=*V_J_*=*K*=*V_K_*) | *O_females(JK)_* | *V_Ofemales(JK)_* | *I_females_* | Parameters | GM | RM | SSM | SSE |  |  |
|  |  |  |  |  |  |  |  |  |  |  |  |
|  | 5 | 0.25 | 619.00 | 9,904.000 | *V_Omales_* | 619.00 | 619.06 | 619.10 | 625.20 |  |  |
|  |  |  |  |  | *I_males_* | 9,904.00 | 9,904.96 | 9,906.32 | 10,003.00 |  |  |
|  |  |  |  |  | *I_males_* / *I_females_* | 1.00 | 1.00 | 1.00 | 1.01 |  |  |
|  |  |  |  |  | *I_average_* | 9,904.00 | 9,904.48 | 9,905.16 | 9,953.50 |  |  |
|  |  |  |  |  |  |  |  |  |  |  |  |
|  | 10 | 1.00 | 9,901.00 | 9,901.000 | *V_Omales_* | 9,901.00 | 9,901.96 | 9,903.30 | 10,000.00 |  |  |
|  |  |  |  |  | *I_males_* | 9,901.000 | 9,901.960 | 9,903.300 | 10,000.000 |  |  |
|  |  |  |  |  | *I_males_* / *I_females_* | 1.00 | 1.00 | 1.00 | 1.01 |  |  |
|  |  |  |  |  | *I_average_* | 9,901.00 | 9,901.48 | 9,902.15 | 9,950.50 |  |  |
|  |  |  |  |  |  |  |  |  |  |  |  |
|  | 15 | 2.25 | 50,121.00 | 9,900.440 | *V_Omales_* | 50,121.00 | 50,125.00 | 50,132.70 | 50,622.20 |  |  |
|  |  |  |  |  | *I_males_* | 9,900.440 | 9,901.400 | 9,902.764 | 9,999.440 |  |  |
|  |  |  |  |  | *I_males_* / *I_females_* | 1.00 | 1.00 | 1.00 | 1.01 |  |  |
|  |  |  |  |  | *I_average_* | 9,900.44 | 9,900.92 | 9,901.60 | 9,949.94 |  |  |
|  |  |  |  |  |  |  |  |  |  |  |  |
