# Supplementary material for: The opportunity for sexual selection and the evolution of non-responsiveness to pesticides, sterility inducers and contraceptives
Source: Heliyon. 2018 Nov 29;4(11):e00943. doi: 10.1016/j.heliyon.2018.e00943 (PMC6275691; doi:10.1016/j.heliyon.2018.e00943)
Supplement: Appendix F [file mmc6.docx]

Appendix F.

The Total Variance in Male Offspring Numbers.

We evaluated the total variance in male offspring numbers for each *JK*-th distribution of female fecundity as,

*V_Omales(JK)_* = Σ *p_q_* (*q V_Ofemales(JK)_*) + Σ *p_q_* (*q O_females(JK)_* – *R O_females(JK)_*)^2^ (F.1)

where *p_q_* equaled the proportion of males in the sample of 100 males belonging to each *q*-th mate number class [in this expression, *p_q_* = *m_q_* / (Σ *m_q_*), where *m_q_* equaled the number of males in each *q*-th mating class], *q* equaled the number of mates males obtained in each mating class, *V_Ofemales(JK)_* equaled the total variance in offspring numbers produced by females as defined in Eq. C.5 above, *O_females(JK)_* equaled the average number of offspring per females as defined in Eq. C.4 above, and *R* equaled the sex ratio as defined above.

As with females, using the variance in male offspring numbers, *V_Omales(JK)_*, and the average offspring number, and recognizing that *O_males(JK)_* = *O_females(JK)_* when the sex ratio is assumed to equal 1, we estimated the opportunity for selection on males for each of the above distributions by dividing the total variance in offspring numbers, *V_OmalesJK)_* by the squared average offspring number, *O_males(JK)_*, or,

*I_males(JK)_* = *V_Omalesl(JK)_* / [*O_males(JK)_*]^2^, (F.2)

where *I_males(JK)_*, equaled the opportunity for selection on males due to their matings with females, producing the *J*-th distribution of litter numbers and the *K*-th distribution of litter sizes, *V_Omales(JK)_* equaled the total variance in offspring numbers for males due to their matings with females producing the *J*-th distribution of litter numbers and the *K*-th distribution of litter sizes, and *O_males(JK)_* equaled the average number of offspring produced by males due to their matings with females producing the *J*-th distribution of litter numbers and the *K*-th distribution of litter sizes (Table 1).
