# Supplementary material for: The opportunity for sexual selection and the evolution of non-responsiveness to pesticides, sterility inducers and contraceptives
Source: Heliyon. 2018 Nov 29;4(11):e00943. doi: 10.1016/j.heliyon.2018.e00943 (PMC6275691; doi:10.1016/j.heliyon.2018.e00943)
Supplement: Appendix G [file mmc7.docx]

Appendix G

The Average Opportunity for Selection within a Population

We considered selection on male and female rats as separate, additive components, in which the strength of selection on each sex is proportional to the fraction of the population consisting of each sex (Shuster and Wade 2003). Thus, we estimated the average opportunity for selection within a particular rat population, *I_average(JK)_*, as the weighted sum of the opportunity for selection operating on each sex or,

*I_average(JK)_* = *p_(males)_* *I_males(JK)_* + *p_(females)_* *I_females(JK)_* (G.1)

where *p_(males)_* equaled the fraction of the population that consists of males, *p_(females)_* equaled the fraction of the population that consists of females [1 – *p_(males)_*] = *p_(females)_*], *I_males(JK)_* equaled the opportunity for selection on males [= *V_Omales(JK)_* / (*O_males(JK)_*)^2^, where *V_Omales(JK)_* and *O_males(JK)_* equaled the variance and average in male offspring numbers, respectively] and *I_females(JK)_* equaled the opportunity for selection on females [= *V_Ofemales(JK)_* / (*O_females(JK)_*)^2^, where *V_Ofemales(JK)_* and *O_females(JK)_* equaled the variance and average in female offspring numbers, respectively]. When the sex ratio is assumed to equal 1, the total opportunity for selection equals the arithmetic average of the opportunity for selection on each sex (Shuster and Wade 2003). However, the above approach allows specific consideration of biases in population sex ratio.

As mentioned, certain rat contraceptives are known to reduce or eliminate male fertility disproportionately to that of females (Mayer et al. 2002, 2004; Dyer et al. 2013, 2014; Pyzyna et al. 2014). While it is possible that such treatments may cause all males in a population to become sterile, it is more likely that some fraction of the male population will consist of non-responders, i.e., males who retain their fertility despite experiencing treatment. The smaller the fraction of nonresponding males, the larger the fraction of males who do not contribute to the next generation at all, and similar to the action of sexual selection, the stronger the opportunity for selection on males will be (Shuster and Wade 2003). Moreover, because in this case, only non-responder males will contribute to the next generation, the opportunity for selection on such a male population represents the opportunity for selection favoring any trait possessed by the favored individuals; thus, in this case, the favored trait is non-responsiveness to the pesticide or sterility-inducing treatment (Shuster and Wade 2003; Krakauer et al. 2011).
