# Supplementary material for: The opportunity for sexual selection and the evolution of non-responsiveness to pesticides, sterility inducers and contraceptives
Source: Heliyon. 2018 Nov 29;4(11):e00943. doi: 10.1016/j.heliyon.2018.e00943 (PMC6275691; doi:10.1016/j.heliyon.2018.e00943)
Supplement: Appendix H [file mmc8.docx]

Appendix H

Assortative Mating and the Evolution of Resistance to Pesticides, Sterility-Inducers and Contraceptives.

Shuster and Wade (2003; p. 180-181; see also Wade and Pruett-Jones 1990; Shuster and Wade 1991) showed that when females mate randomly with replacement, 1/Nmales describes a female’s tendency to mate with a given male, and (1 – 1/ Nmales) describes a females tendency not to mate with that male. If the magnitude of a female’s tendency to mate non-randomly is represented by s, then a female’s probability of mating with a preferred male is increased above 1/ Nmales by s, and her probability of mating with an un-preferred male is decreased below 1/ Nmales by s. This pattern of female preference creates two classes of males, males who succeed in mating and males who fail to mate. When some males are excluded from mating by the mating success of other males, sexual selection occurs (Darwin 1871; Shuster and Wade 2003).

The opportunity for sexual selection on males arising under this form of nonrandom mating, Imates(preferred) is increased over the opportunity for sexual selection that arises on males as a result of random mating, Imates(random), by the degree to which female preferences influence the proportions of the male population that consist of mating and non-mating males, thus,

Imates(preferred) = [Imates(random)] [Nmales + Nfemales (s)] / [Nmales + s] (H.1)

where Imates(preferred) equals the opportunity for sexual selection on males when female mate choice is non-random, Imates(random) equals the opportunity for sexual selection when female mate choice is random, Nmales and Nfemales equal the number of males and females in the population, respectively, and s equals the magnitude of the preference females have for particular mates (Shuster and Wade 2003, p. 181). The effect of different values of s on the magnitude of Imates(preferred) is shown in more detail elsewhere (Wade and Pruett-Jones 1990; Shuster and Wade 1991). Note that the notation “Imates” refers to the sex difference in the opportunity for selection, or the opportunity for sexual selection, arising as a result of differential mating success among males (Shuster and Wade 2003). The term Imates is expressed elsewhere as Is (Jones 2009).

References

Shuster, S.M., Wade, M.J., 1991. Female copying and sexual selection in a marine isopod crustacean. Anim. Behav. 42, 1071–1078.

Wade, M.J., Pruett-Jones, S., 1990. Female copying increases the variance in male mating success. Proc. Natl. Acad. Sci. U. S. A. 87, 5749–5753.
